# Genome-wide sequencing as a first-tier screening test for short tandem repeat expansions

**DOI:** 10.1186/s13073-021-00932-9

**Published:** 2021-08-09

**Authors:** Indhu-Shree Rajan-Babu, Junran J. Peng, Readman Chiu, IMAGINE Study, Shelin Adam, Christele Du Souich, Alison Elliott, Anna Lehman, Jill Mwenifumbo, Tanya Nelson, Clara van Karnebeek, Jan Friedman, CAUSES Study, Patricia Birch, Madeline Couse, Colleen Guimond, Anna Lehman, Jill Mwenifumbo, Clara van Karnebeek, Jan Friedman, Chenkai Li, Arezoo Mohajeri, Egor Dolzhenko, Michael A. Eberle, Inanc Birol, Jan M. Friedman

**Affiliations:** 1 Department of Medical Genetics, University of British Columbia and Children’s & Women’s Hospital, Vancouver, BC V6H3N1 Canada; 2 Department of Medical and Molecular Genetics, King’s College London, Strand, London, WC2R 2LS UK; 3 Canada’s Michael Smith Genome Sciences Centre, BC Cancer Agency, Vancouver, BC V5Z4S6 Canada; 4 Bioinformatics Graduate Program, University of British Columbia, Vancouver, BC V6T1Z4 Canada; 5 Illumina Inc., San Diego, CA 92121 USA

**Keywords:** Clinical bioinformatics, Repeat expansion, Next-generation sequencing, Short tandem repeats, Machine learning

## Abstract

**Background:**

Screening for short tandem repeat (STR) expansions in next-generation sequencing data can enable diagnosis, optimal clinical management/treatment, and accurate genetic counseling of patients with repeat expansion disorders. We aimed to develop an efficient computational workflow for reliable detection of STR expansions in next-generation sequencing data and demonstrate its clinical utility.

**Methods:**

We characterized the performance of eight STR analysis methods (lobSTR, HipSTR, RepeatSeq, ExpansionHunter, TREDPARSE, GangSTR, STRetch, and exSTRa) on next-generation sequencing datasets of samples with known disease-causing full-mutation STR expansions and genomes simulated to harbor repeat expansions at selected loci and optimized their sensitivity. We then used a machine learning decision tree classifier to identify an optimal combination of methods for full-mutation detection. In Burrows-Wheeler Aligner (BWA)-aligned genomes, the ensemble approach of using ExpansionHunter, STRetch, and exSTRa performed the best (precision = 82%, recall = 100%, F1-score = 90%). We applied this pipeline to screen 301 families of children with suspected genetic disorders.

**Results:**

We identified 10 individuals with full-mutations in the *AR*, *ATXN1*, *ATXN8*, *DMPK*, *FXN*, or *HTT* disease STR locus in the analyzed families. Additional candidates identified in our analysis include two probands with borderline *ATXN2* expansions between the established repeat size range for reduced-penetrance and full-penetrance full-mutation and seven individuals with *FMR1* CGG repeats in the intermediate/premutation repeat size range. In 67 probands with a prior negative clinical PCR test for the *FMR1*, *FXN*, or *DMPK* disease STR locus, or the spinocerebellar ataxia disease STR panel, our pipeline did not falsely identify aberrant expansion. We performed clinical PCR tests on seven (out of 10) full-mutation samples identified by our pipeline and confirmed the expansion status in all, showing absolute concordance between our bioinformatics and molecular findings.

**Conclusions:**

We have successfully demonstrated the application of a well-optimized bioinformatics pipeline that promotes the utility of genome-wide sequencing as a first-tier screening test to detect expansions of known disease STRs. Interrogating clinical next-generation sequencing data for pathogenic STR expansions using our ensemble pipeline can improve diagnostic yield and enhance clinical outcomes for patients with repeat expansion disorders.

**Supplementary Information:**

The online version contains supplementary material available at 10.1186/s13073-021-00932-9.

## Background

Expansions of short tandem repeats (STRs; tandemly repeated arrays of 1–6 base pair (bp) sequence motifs [1]) can cause several neurological and neuromuscular disorders [2]. Accurate genotyping (i.e., the determination of the number of copies of repeat units in an STR) is critical to the molecular diagnosis of STR expansion disorders as repeat length often influences disease severity and age of onset of clinical symptoms [3]. Repeat length also determines an STR’s allelic class (normal, intermediate, premutation, or full-mutation), which may differ with respect to the associated disease phenotype [3, 4]. For example, the *FMR1* (MIM 309550) premutation (55–200 CGG repeats) increases the risk for primary ovarian insufficiency (MIM 311360) and tremor/ataxia syndrome (MIM 300623). In contrast, *FMR1* full-mutation (> 200 CGG repeats) causes fragile X syndrome (MIM 300624), the most frequent Mendelian cause of intellectual disability [5]. Premutation and intermediate (also known as “mutable normal”) alleles that are meiotically unstable can expand into pathogenic full-mutation in a single generation, while normal alleles rarely, if ever, do so [6, 7]. Expanded alleles tend to further increase in repeat length during intergenerational transmission, and as a result, genetic anticipation (the earlier and more severe manifestation of disease symptoms with each successive generation) is common in repeat expansion disorders [8].

Clinical laboratories typically use polymerase chain reaction (PCR) or Southern blot (alone or in combination) to characterize expansions at known disease STR loci [9]. Although highly sensitive in detecting and genotyping STR expansions, PCR and Southern blot tests have several limitations. They are time- and labor-intensive, require extensive optimization, and do not permit concurrent analyses of more than a handful of STR loci. Next-generation sequencing (NGS), on the other hand, enables exome- or genome-wide characterization of STRs.

Several algorithms have recently been developed to analyze STRs in NGS data [1, 10–14]. The incorporation of bioinformatics tools to screen for STR expansions may permit the diagnosis of repeat expansion disorders during routine diagnostic exome or genome sequencing, allowing accurate genetic counseling of affected individuals and their families, and improving clinical outcomes. The currently available STR analysis algorithms have different attributes that determine their utility and sensitivity in detecting and characterizing repeat expansions in NGS data (Table 1). Methods like STRetch [11] and exSTRa [12] identify STR expansions via case-control analysis, with a caveat of either underestimating the repeat lengths of some expanded STRs [11] or not providing repeat length estimates at all [12]. Methods that genotype STRs perform better across certain repeat length ranges depending on the read type evidence considered. For instance, lobSTR [15], HipSTR [16], and RepeatSeq [17], which only rely on reads that fully encompass an STR (“spanning reads”) to compute repeat length, can size alleles within the length of an Illumina read (125–150 bp) but perform poorly in detecting pathogenic full-mutation expansions that exceed read length. More recent methods [1, 10, 13, 14] that leverage additional read types such as flanking or partially flanking reads (those that map to unique flanking sequences), in-repeat reads (IRR; those that are entirely composed of STRs with a read-pair mate that maps to the STR’s flanking sequence), and/or IRR pairs (both reads of a pair mapping to the STR) can size STRs that exceed read length. ExpansionHunter (EH) [10, 13] and GangSTR [14], in particular, enable the recovery of IRR and IRR pairs, which originate from an expanded STR but may incorrectly map to other STR (or “off-target”) regions with longer tracts of the same repeat motif. By allowing the inclusion of off-target sites (OTS), EH and GangSTR analyses facilitate sizing STRs that are longer than an Illumina sequencing library fragment length (350–500 bp).

**Table 1:** Features of some publicly available short tandem repeat analysis algorithms

Features	lobSTR	RepeatSeq	HipSTR	TRED	EH	STRetch	exSTRa	GangSTR
Outputs repeat length?	Y	Y	Y	Y	Y	Y	N	Y
Sequencing reads	Single- and paired-end	Single- and paired-end	Single- and paired-end	Paired-end	Paired-end	Paired-end	Paired-end	Paired-end
Sequencing platforms supported	Illumina, Sanger, 454, and IonTorrent	Illumina	Illumina	Illumina	Illumina	Illumina	Illumina	Illumina
Library prep. supported	PCR and PCR-free	n.a.	PCR and PCR-free	PCR and PCR-free	PCR and PCR-free	PCR and PCR-free	PCR and PCR-free	PCR and PCR-free
Library prep. (rcmd)	None	None	None	None	PCR-free	PCR-free	None	None
Aligners (rcmd)	lobSTR and BWA-MEM	Novoalign and Bowtie 2	Indel-sensitive aligner	None	None	None	Bowtie 2	None
Analysis approach	Targeted and GW	Targeted and GW	Targeted and GW	Targeted	Targeted	GW	Targeted and GW	Targeted and GW
NGS data type supported	WGS	WGS	WGS	WGS	WGS and ES	WGS and ES	WGS and ES	WGS and ES
NGS data format	.bam, .fastq, or .fasta	.bam	.bam	.bam	.bam or .cram	.bam or .fastq	.bam	.bam
Built-in stutter correction model^a^	Y	Y	Y	Y	n.a.	n.a.	n.a.	Y
Test of significance	N	N	N	N	N	Y	Y	N
Read types used	Spanning	Spanning	Spanning	Spanning, flanking or partial, paired-end reads, and IRR	Spanning, flanking, and IRR/IRR pairs	Anchored IRR	Flanking and anchored IRR	Spanning, flanking, and IRR/IRR pairs
Phasing^b^	n.a	n.a	Y	n.a	n.a	n.a	n.a	n.a
PL	C++	C++	C++	Python	C++	Java	Perl and R	C++
Sizing limitation	RL	RL	RL	FL	Not limited	FL	n.a.	Not limited
Control dataset	Not required	Not required	Not required	Not required	Not required	Required	Not required	Not required
Complex repeats	n.a.	n.a.	n.a.	n.a.	Y	n.a.	n.a.	N
Output files	.vcf and .allelotype.stats	.repeatseq, .calls, and .vcf	.vcf	.vcf and .json	.vcf, .json, and .log	.tsv	p-values, ECDF, and tsum plots	.vcf
Customized regions file	Possible	Possible	Possible	Possible	Possible	Possible, but not rcmd	Possible	Possible

*EH* ExpansionHunter, *TRED* TREDPARSE, *Y* feature included, *N* feature not included, *Library prep* library preparation protocol, *rcmd* recommended, *PL* programming language used, *n.a.* not applicable/information not available, *GW* genome-wide, *WGS* whole-genome sequencing, *ES* exome sequencing, *IRR* in-repeat reads, *RL* read length, *FL* fragment length, *Not limited* not limited by either RL or FL, *ECDF* Empirical Cumulative Distribution Function, *t-sum* aggregated T statistic

^a^Corrects the noise (stutters) introduced during PCR amplification-based library preparation

^b^Utilizes phased single nucleotide variant haplotypes

In terms of utility, some of these methods can analyze STRs in both exome sequencing (ES) and whole-genome sequencing (WGS) data [11, 12, 14], while others are designed specifically for WGS [1, 10, 13]. Some tools have specific NGS data requirements; for example, EH is designed for PCR-free WGS, and exSTRa has only been extensively tested on bowtie 2 [18] alignments. Also, most methods perform less well on GC-rich STR expansions [10, 12].

These varied attributes and performance characteristics have led to the acknowledgement that a single bioinformatics tool is less likely to be able to identify pathogenic STR expansions of all repeat lengths and sequence content/composition in NGS data [12]. Recently, Tankard et al. recommended a consensus calling approach using at least two out of four tools (TREDPARSE [1], EH, STRetch, and exSTRa) to characterize expansions of known disease STRs [12]. However, it is not clear which of these (or other) STR methods alone or in combination yield optimal sensitivity and specificity.

In this study, we employed a decision tree classifier to identify the optimal tool(s) for classifying expanded full-mutation and non-expanded alleles at known disease STR loci with high accuracy, precision, recall, and F1-score. We performed our analysis on STR calls from eight different tools (lobSTR, HipSTR, RepeatSeq, EH, TREDPARSE, GangSTR, STRetch, and exSTRa) made on WGS data of patients with well-characterized STR expansions in one of eight different loci (*AR*, *ATN1*, *ATXN1*, *ATXN3*, *DMPK*, *FMR1*, *FXN*, or *HTT*) and simulated WGS data harboring expansions at GC-rich *FMR1*, *FMR2*, or *C9orf72* STR loci. We included the spanning-read-only algorithms (lobSTR, HipSTR, and RepeatSeq), which have been used in recent studies to characterize normal polymorphic STR variations [19, 20], to investigate their utility and reliability in genotyping alleles that are within read length compared to more recent algorithms that consider a wider variety of read type evidence. The WGS data were aligned using two different aligners, Isaac [21] — an ultra-fast aligner, and Burrows-Wheeler Aligner (BWA)-MEM [22] — which is widely used in WGS studies [23], to see if the choice of aligner would influence the performance of STR methods.

First, we tested the classifier on results generated by the implementation of tools using default parameter settings. We then tweaked several parameters to optimize the sensitivity and specificity of STR tools included in this study. Once we established the parameters that yielded the best results, we used data generated with these settings to train and test the classifier and found a significant improvement in our model’s ability to detect full-mutations compared to our default assessment. We then applied our decision tree model of STR algorithms to screen for expansions in known disease STR loci in ES and WGS data of 301 families (patient-parent trios (patient and both biological parents) or quads (patient, similarly affected sibling, and both biological parents)) with a proband who is suspected clinically to have a genetic disorder.

## Methods

### WGS datasets with a known repeat expansion

The WGS datasets with a known repeat expansion analyzed in this study include the BWA and Isaac alignments of: (1) the European Genome-phenome Archive (EGA) dataset [10] (EGAD00001003562), which consisted of data from 118 PCR-free WGS of Coriell samples, each with an *AR*, *ATN1*, *ATXN1*, *ATXN3*, *DMPK*, *FMR1*, *FXN*, or *HTT* expansion (see Additional file 1: Table S1), and (2) *C9orf72, FMR1,* or *FMR2* expansions of varying repeat lengths simulated using the ART NGS read simulator [24] (Additional file 1: Table S2). The simulated WGS data were included in our analysis to assess the performance of STR callers on expansions of extremely high GC content (100%) that may be refractory to detection.

### Patient cohorts and ES and WGS data generation

The patient cohorts screened for known STR expansions in this study consist of the ES data of 141 trios or quads from the Clinical Assessment of the Utility of Sequencing and Evaluation as a Service (CAUSES) Study [25] and the WGS data of 160 trios or quads from the Integrated Metabolomics And Genomics In Neurodevelopment (IMAGINE) [26] or CAUSES Studies. Subjects enrolled in the CAUSES Study were children who were suspected on clinical grounds to have a single gene disorder but in whom conventional testing had not identified a genetic cause. Subjects enrolled in the IMAGINE Study had impairment of motor function with onset before birth or within the first year of life and additional clinical features that made perinatal complications such as hypoxia or intracranial hemorrhage an unlikely explanation for their symptoms. The CAUSES and IMAGINE Study subjects were enrolled at the Children’s and Women’s Health Centre (Vancouver, British Columbia, Canada), and most of the enrolled subjects in both studies had intellectual disability. The ES or WGS data from the unaffected parents were used to verify the inheritance or unstable transmission of variants. The CAUSES and IMAGINE Studies were approved by the Institutional Review Board of the Children’s and Women’s Health Centre of British Columbia and the University of British Columbia (H15-00092 and H16-02126, respectively).

The trio/quad ES data were sequenced by Ambry Genetics (Aliso Viejo, USA), Centogene (Rostock, Germany), or Canada’s Michael Smith Genome Sciences Centre (Vancouver, Canada) to a mean coverage of ~ 60×. The library preparation protocols and sequencers used to generate the trio/quad ES data are described in Additional file 2: Table S3.

The median coverage of the trio/quad WGS data ranged from 36 to 80× and was generated by the McGill University and Genome Quebec Innovation Centre (Quebec, Canada) or Canada’s Michael Smith Genome Sciences Centre. WGS libraries were prepared using the NxSeq® AmpFREE Low DNA Library Kit Library Preparation Kit and Adaptors (Lucigen, Wisconsin, US) or xGen Dual Index UMI Adapters (Integrated DNA Technologies, Coralville, US) and sequenced on an Illumina HiSeqX sequencer.

The paired-end reads (125 or 150 bp) of both ES and WGS datasets were aligned to the UCSC hg19 human reference genome using BWA-MEM, and duplicates were marked with Picard [27]. All patient ES data underwent single-nucleotide variant (SNV) and indel analysis, and 140 out of the 141 trios or quads included in this study had no clinically relevant SNV/indel variants. We also analyzed the ES data of a quad with known myotonic dystrophy (type 1; DM1–MIM 160900) in the proband and his mother as a positive control. Our patient WGS data underwent SNV, indel, structural, and mitochondrial variant analysis. We have diagnosed a genetic disease in 44 of 85 (52%) trios/quads studied in the CAUSES Study. Similarly, we have diagnosed a genetic disease in 46 of 88 (52%) trios/quads studied in the IMAGINE Study to date. The WGS data of all trios/quads from both CAUSES and IMAGINE Studies were included in this study.

### Bioinformatics tools for STR analysis

The STR analysis tools implemented in this study include lobSTR [15], HipSTR [16], RepeatSeq [17], TREDPARSE [1], EH [10, 13], GangSTR [14], STRetch [11], and exSTRa [12]. The key features of these tools and the commands and parameters used to execute them are described in Table 1 and Additional file 2: Table S4, respectively. The two versions of EH v2 [10] and v3 [13] implement different algorithms. While we found EH_v2 to be easily tailorable for other STR loci, EH_v3 facilitates the analysis of STRs with complex or mixed repeat motifs. Therefore, we evaluated both versions of EH.

### Disease STR catalogs

The STR analysis tools assess known disease STRs included within a pre-defined STR catalog that contains repeat coordinate/motif information supplied by the tools’ developers. The known pathogenic STR loci included in these catalogs, as well as their allelic categories and corresponding repeat lengths, are summarized in Additional file 2: Table S5. Notably, the region files for EH only included pre-defined OTS for *FMR1* and *C9orf72* loci, while GangSTR included OTS in the region files of all 12 pathogenic STR loci provided with the tool. Some of the known disease STRs analyzed in this study (*AR*, *ATN1*, *FXN*, and *FMR2*) were missing for GangSTR. Therefore, we added these loci and included their OTS as described in Mousavi et al. [14].

### Interpretation of full-mutations and non-full-mutations

The data from the genotyping methods were classified as “full-mutation” if the estimated repeat lengths of the STRs exceeded their respective full-mutation thresholds (Additional file 2: Table S5). STRetch and exSTRa calls were classified as “full-mutation” if the *p*-values post-multiple-testing-adjustment were less than < 0.05. For STRetch, we used the control file (containing data from 143 healthy individuals) provided with the tool.

### Negative control cohort

Our negative control cohort included 100 individuals (healthy parents of affected children) from the IMAGINE Study who did not harbor expanded disease STR alleles based on the genotype calls from TREDPARSE, EH, and GangSTR.

### Decision tree classification

Decision tree analysis is a supervised machine learning classification method [28]. We employed this approach to infer the best model or best combination of STR analysis tools to detect full-mutation expansions with optimal sensitivity and specificity. We used the Python scikit-learn machine learning library [29] to implement the decision tree classifier and used STR calls from EGA WGS to train and test the classifiers on data from Isaac and BWA alignments.

For our preliminary decision tree analysis, we used the outputs generated using the default parameters for each of the STR analysis tools. We compiled the results generated by the STR analysis tools on Isaac- and BWA-aligned WGS data. We labeled each EGA genome’s true STR expansion status or class label as full-mutation or non-full-mutation for a given locus. The single known or characterized STR expansion in each of the EGA and simulated genomes was assigned to the “full-mutation” class, while the status of the other STR loci was assigned to “non-full-mutation”. The data from STR callers were then transformed into binary flags: 1 indicating at least one of the two alleles was called as “full-mutation”, and 0 indicating both alleles were “non-full-mutation”. From there, we removed all rows with missing values and supplied the data to the classifier. We divided our dataset into 80 and 20% to train and test the classifier, respectively.

We used the Gini index approach to ascertain the efficiency of an attribute (i.e., the STR caller) in differentiating samples belonging to the full-mutation and non-full-mutation classes. To evaluate the performance of the classifier, we extracted different metrics, including precision (true positives [TP]/(TP + false positives [FP])), recall (TP/(TP + false negatives [FN])), accuracy ((TP + true negatives [TN])/(TP + TN + FP + FN)), and F1-score (2 × ((precision × recall)/(precision + recall))), and analyzed the receiver operating characteristic (ROC) curve, a ratio of sensitivity (TP/(TP + FN)) and inverted specificity (1 − (TN/(TN + FP))), and precision-recall curve, a ratio of precision and recall or sensitivity.

To identify the best analysis model, we first selected important features using the Exhaustive Feature Selector algorithm from the machine learning extensions (MLxtend) Python library [30], which assessed the performance of all possible combinations of STR tools on 50 train-validation splits generated by five repeats of stratified 10-fold cross-validation on the training dataset. We then chose the best feature subset with the highest mean ROC_AUC (area under the curve) and tuned the hyper-parameters of the decision tree using the scikit’s GridSearchCV on the training dataset for improved predictions.

We next ascertained whether tweaking some of the parameters would improve the performance of the STR analysis tools and the resultant decision tree model. First, we assessed the performance of EH with OTS on selected STR loci that are known to harbor expansions exceeding sequencing fragment lengths to retrieve unmapped and mismapped IRR/IRR pairs and improve the repeat length estimation and detection of full-mutations. Second, we used the intermediate repeat length threshold instead of the full-mutation threshold for the *FMR1* locus to classify expanded alleles and documented the sensitivity as well as the false-positive rates of the genotypers. Third, we tested exSTRa’s performance with control data from our negative control cohort. We carefully evaluated how these parameter tweaks influenced the performance of the STR analysis tools and selected the optimized outcomes to reimplement our decision tree classification analysis as mentioned above, and compared the results to our preliminary decision tree analysis with default parameters.

### Screening for known disease STR expansions in patient data

Finally, we screened our patient trio/quad ES and WGS data for known disease STR expansions using the tools identified by the classifier. Of the probands analyzed in this study, 60 have had a clinical *FMR1* STR testing, three have had clinical spinocerebellar ataxia (SCA) STR panel tests, one has had a clinical *FXN* STR test, and four have had a clinical *DMPK* STR test. All of these clinical PCR-based STR tests were negative for a pathogenic expansion, except for a confirmed *DMPK* full-mutation in a proband and his mother. All individuals who were expansion-negative at the tested locus were used as negative controls.

For all expanded STRs identified in patients, we analyzed the parental genotype calls to verify the inheritance or unstable transmission of the alleles. Subjects with potential expansions of known disease STRs were identified for orthogonal validation to ascertain the specificity of our decision tree. Molecular testing (PCR and capillary electrophoresis) of some of the identified STR candidates was performed by Centogene (Germany).

## Results

### Performance of STR algorithms on Isaac- versus BWA-aligned WGS data

The lobSTR [15], HipSTR [16], RepeatSeq [17], EH versions 2 [10] and 3 [13], GangSTR [14], TREDPARSE [1], STRetch [11], and exSTRa [12] results of Isaac- and BWA-aligned EGA and simulated *FMR2* and *C9orf72* WGS data are shown in Additional file 2: Table S6 and S7, respectively.

Of the known normal alleles (n = 94) in EGA/simulated Isaac WGS, GangSTR, TREDPARSE, EH_v2, and EH_v3 correctly genotyped > 89% of normal alleles (Additional file 2: Table S8). lobSTR, HipSTR, and RepeatSeq, on the other hand, correctly genotyped ~ 30–75% of normal alleles; however, these tools were generally inconsistent in genotyping normal alleles of the *C9orf72*, *FMR1*, *FMR2*, *FXN*, and/or *HTT* loci. Of the known *FMR1* intermediate and premutation alleles (n = 21), EH_v2 and EH_v3 correctly identified 18 and 16 alleles, respectively, followed by TREDPARSE (eight alleles) and GangSTR (six alleles) (Additional file 2: Table S9). lobSTR, HipSTR, and RepeatSeq either under-sized all known intermediate/premutation alleles or did not genotype them. We observed a similar trend among genotyped normal and intermediate/premutation alleles in BWA data, except that GangSTR did not correctly identify any intermediate/premutation alleles. As they only rely on spanning reads, lobSTR, HipSTR, and RepeatSeq did not detect any full-mutations in either Isaac- or BWA-aligned WGS data.

The sensitivity of EH_v2, EH_v3, GangSTR, TREDPARSE, STRetch, and exSTRa run with default parameters to detect full-mutations across the different analyzed STR loci in Isaac- and BWA-aligned WGS is summarized in Table 2. EH_v2, EH_v3, TREDPARSE, and STRetch exhibited consistent performance, with a sensitivity of ~ 70% in both Isaac and BWA alignments. GangSTR’s sensitivity was better on Isaac (55%) compared to BWA (38%) alignments. In marked contrast, exSTRa detected more full-mutations for BWA (88%) alignments compared to Isaac (56%) (Additional file 3: Fig S1 for exSTRa’s plots on Isaac- and BWA-aligned WGS). On Isaac-aligned data, STRetch, EH_v2, and EH_v3 detected the most full-mutations, followed by TREDPARSE, exSTRa, and GangSTR. On BWA-aligned data, exSTRa detected the most full-mutations, followed by STRetch, EH_v2, EH_v3, TREDPARSE, and GangSTR. Notably, although exSTRa and STRetch detected more full-mutations, they also had the most false-positive calls.

**Table 2:** Full-mutation samples detected in the EGA and simulated genomes using the default implementation of STR analysis tools

Gene	***AR*** (n = 1)	***ATN1*** (n = 2)	***ATXN1*** (n = 3)	***ATXN3*** (n = 1)	***C9orf72*** (n = 3)	***DMPK*** (n = 17)	***FMR1*** (n = 18)	***FMR2*** (n = 3)	***FXN*** (n = 25)	***HTT*** (n = 13)	FPs	Total FM detected	Sensitivity
FM threshold (rpts)	37	47	38	59	60	50	200	200	65	39			
Allelic classification	FM	FM	FM	FM	FM	FM	FM	FM	NL/FM or FM/FM	NL/FM or FM/FM			
**Isaac**													
EH_v2	1	2	2	1	3	17	1	0	25	13	6	65	0.75581395
EH_v3	1	2	3	0	3	17	0	0	25	13	5	64	0.74418605
GangSTR	0	2	2	0	0	16	0	0	16	11	8	47	0.54651163
TRED	1	2	1	0	3	17	0	0	25	13	3	62	0.72093023
STRetch	1	2	3	1	3	17	2	3	20	13	26	65	0.75581395
exSTRa	1	2	3	0	3	17	1	3	5	13	33	48	0.55813953
**BWA**													
EH_v2	1	2	2	1	3	17	0	0	25	13	6	64	0.74418605
EH_v3	1	2	3	0	3	17	0	0	25	13	5	64	0.74418605
GangSTR	1	2	2	1	1	16	0	0	0	10	8	33	0.38372093
TRED	1	2	1	0	3	17	0	0	25	13	10	62	0.72093023
STRetch	1	2	3	1	3	17	2	3	20	13	26	65	0.75581395
exSTRa	1	2	3	1	3	16	9	3	25	13	35	76	0.88372093

The analyzed dataset had 86 samples with at least one known full-mutation allele. The number of true-positives detected by the tools, sensitivity, and the number of false positives identified in our default analysis of the Isaac- (top panel) and BWA-aligned (bottom panel) genomes are shown. *NL* normal, *FM* full-mutation, *FPs* false-positives, *rpts* repeats, *EH_v2* ExpansionHunter version 2, *EH_v3* ExpansionHunter version 3, *TRED* TREDPARSE

All full-mutations missed by genotypers were under-sized and classified incorrectly as premutation, intermediate, or normal (Additional file 2: Table S10). Among the analyzed STR loci, *FMR1*, *FMR2*, and homozygous *FXN* full-mutations were particularly refractory to detection.

### Simulated versus real genomes

We next implemented the STR callers on *FMR1* simulations (13 BWA-aligned genomes simulated to harbor normal–full-mutation *FMR1* alleles) with repeat counts similar to those of the fragile X reference samples in the EGA dataset to investigate whether GC-bias in experimental WGS is reflected in simulated data as well (Additional file 2: Table S11). All STR callers performed significantly better on simulated WGS with premutation and full-mutation *FMR1* expansions compared to real data. Upon investigating the read-support evidence from EH, we found that simulated WGS with premutation and full-mutation expansions had more IRRs in comparison to real WGS, demonstrating that simulated data of GC-rich repeat expansions could artificially inflate the accuracy and performance of STR callers. Subsequently, we excluded simulated genomes from the analysis.

### Decision tree classification

We first trained and tested the decision tree classifier on the generated default parameter results of EH_v2, EH_v3, GangSTR, TREDPARSE, STRetch, and exSTRa in the EGA genomes. After removing the rows with missing values, the compiled STR calls of Isaac- and BWA-aligned WGS datasets had 1178 and 1176 rows (one row per sample per STR locus), respectively. We identified EH_v2, GangSTR, and exSTRa as the best feature set in Isaac data, tuned the hyper-parameters of the classifier with selected features, and implemented the model with the best hyper-parameters and features on test data. Additional file 3: Fig S2 shows the decision tree with EH_v2, which performed the best (had the lowest Gini impurity) in classifying alleles, assigned to the root node (node #0). In the test data, the decision tree model had precision, recall, and F1-score of 100, 83, and 91%, respectively, to detect full-mutations; for non-full-mutations, the precision, recall, and F1-score were 99, 100, and 99%, respectively (Additional file 3: Fig S3a). The ROC and precision-recall plots are shown in Additional file 3: Fig S3b and the confusion matrix showing the classification of full-mutations and non-full-mutations in the test dataset is shown in Additional file 3: Fig S3c.

In BWA-aligned data, the best model included EH_v3, STRetch, exSTRa, GangSTR, and TREDPARSE. EH_v3 at the root node correctly classified 45 out of 62 full-mutations and 873 out of 878 non-full-mutations in the training dataset, with exSTRa and GangSTR recovering one of the full-mutations missed by EH_v3 (Additional file 3: Fig S4). The precision, recall, and F1-score to detect full-mutations and non-full-mutations in the test data were 100, 89, and 94% and 99, 100, and 100%, respectively (Additional file 3: Fig S5a). The ROC and precision-recall curves and confusion matrix are shown in Additional file 3: Fig S5b and S5c.

In Isaac and BWA analyses, three and five of the six features (i.e., STR tools) contributed to the performance of the model, led by EH_v2 and EH_v3, respectively (Additional file 3: Fig S3d and S5d). The sensitivity for detecting full-mutations in BWA-aligned data was slightly higher compared to the Isaac analysis. Overall, the decision tree classifier on Isaac and BWA test data using the default analysis missed ~ 10 to 20% of full-mutations. To improve the detection sensitivity, we evaluated some parameters that we believed might help capture more of the true full-mutations.

#### Tested parameters

First, we tested the effect of including OTS in the detection of full-mutations. While GangSTR’s region files included OTS for all analyzed loci, the author-supplied JSON files of EH did not include OTS for *DMPK* and *FXN* loci, which are known to harbor expansions exceeding fragment lengths. In our initial EH run without OTS, we noted reduced sensitivity in the detection of *FXN* full-mutations (Additional file 2: Table S12). Therefore, we added OTS for analyzing these loci with EH_v2. There was no improvement in sensitivity, highlighting the general limitation of the genotypers in reliably detecting homozygous *FXN* full-mutation expansions.

Second, because GC-rich expansions such as those at the *FMR1* locus tend to be under-sized owing to reduced coverage even in PCR-free Illumina WGS datasets [10], we used an intermediate repeat length threshold (54 repeats) for *FMR1* instead of their full-mutation threshold (200 repeats). With this tweak, EH_v2 and EH_v3 detected all *FMR1* full-mutations in Isaac- as well as BWA-aligned data (Table 3). TREDPARSE detected 83–89% of *FMR1* full-mutations, while GangSTR detected 16–22% of them. The identified false positives in this analysis include the known *FMR1* premutations and a few borderline *FMR1* intermediate alleles that are closer to the threshold.

**Table 3:** *FMR1* and *FMR2* full-mutations detected by ExpansionHunter, GangSTR, and TREDPARSE with lowered repeat length threshold

Aligner	Isaac							BWA						
Locus	*FMR1* (n = 18)				*FMR2* (n = 3)			*FMR1* (n = 18)				*FMR2* (n = 3)		
FM threshold	54 repeats				60 repeats			54 repeats				60 repeats		
Allelic classification	FM	IM	NL	FP	FM	NL	FP	FM	IM	NL	FP	FM	NL	FP
EH_v2	18	.	.	20	3	.	0	18	.	.	16	3	.	0
EH_v3	18	.	.	22	3	.	0	18	.	.	22	3	.	0
GangSTR	4	.	14	7	0	3	0	3	.	15	0	0	3	0
TREDPARSE	15	1	2	8	0	3	0	16	.	2	13	0	3	0

The number of full-mutations (FMs) misclassified as normal (NL; < 45 repeats for *FMR1* and < 31 repeats for *FMR2*) or intermediate (IM; 45–54 repeats for *FMR1*) allele are shown. The true number (n) of known FM alleles in the *FMR1* and *FMR2* genes is indicated in parenthesis. False-positive (FP) calls made by the tools are also reported

Lastly, we hypothesized that adding data from a control cohort to exSTRa’s analysis would further improve its full-mutation detection sensitivity. In Isaac alignments, exSTRa’s sensitivity improved moderately for detecting *FXN* full-mutations, but no improvements were observed in full-mutation detection at other loci (Additional file 3: Fig S6a). In BWA alignments, exSTRa yielded better sensitivity with controls and detected all homozygous *FXN* full-mutation expansions, as well as all *FMR1* full-mutations (Additional file 3: Fig S6b).

**Fig. 1:**
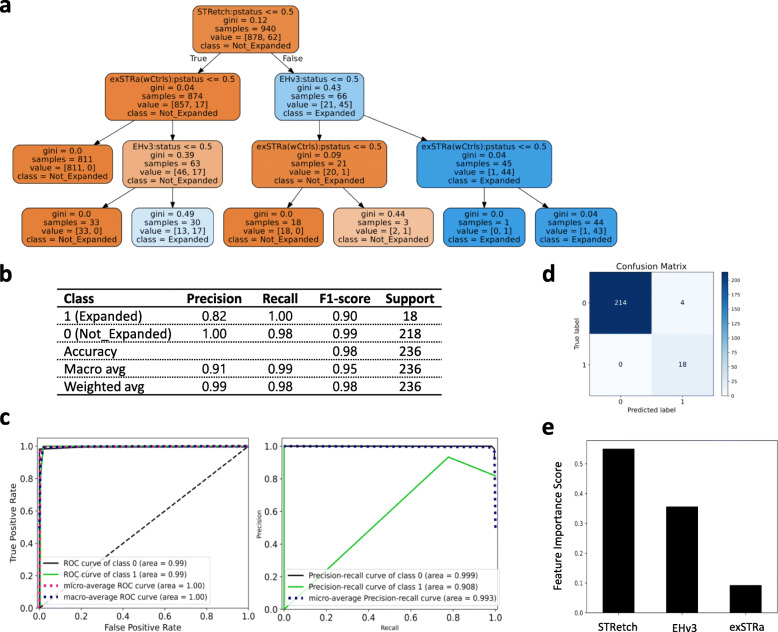
Decision tree model and its performance metrics on modified analysis of BWA-aligned EGA genomes. **a** Decision tree generated on the training dataset (*n* = 940). Node #0 at the top of the tree is the root node. Each node lists an STR tool (feature). The “samples” number represents the total number of genotype calls in a particular node, and “value” shows the number of expanded (or full-mutation, FM) and non-expanded (non-FM) genotypes. Gini index shows the impurity at each node. The terminal nodes or leaves with a Gini value of 0 have genotypes belonging entirely to either the expanded or non-expanded class. EHv3, ExpansionHunter version 3; wCtrls, analysis performed with controls. **b** Classification report summarizing the performance metrics of the model on test data (*n* = 236). Macro and weighted average (avg) show the unweighted and weighted mean of performance metrics calculated for Expanded and Not_Expanded class labels, respectively. **c** Receiver operating characteristics and precision-recall curves. **d** Confusion matrix showing the number of predicted and true labels on *x*- and *y*-axis, respectively. **e** Feature importance plot showing the STR tool on *x*-axis and the tool’s normalized (Gini) importance on *y*-axis

Of these parameters, using the intermediate threshold for *FMR1* genotype analysis and performing exSTRa’s analysis with controls were useful in detecting refractory STR expansions. We provided these improved results to the classifier. In Isaac data, feature selection and hyperparameter tuning helped identify TREDPARSE, STRetch, and EH_v3 as the best feature subset (Additional file 3: Fig S7). In BWA data, the best feature subset included STRetch, EH_v3, and exSTRa (Fig. 1a). The classifier’s precision, recall, and F1-score in Isaac- and BWA-aligned test datasets were 90, 100, and 95% and 82, 100, and 90% to detect full-mutations and 100, 99, and 100% and 100, 98, and 99% to detect non-full-mutations, respectively (Additional file 3: Fig S8a, and Fig. 1b). The ROC and precision-recall curves are shown in Additional file 3: Fig S8b and Fig. 1c, and the confusion matrix in Additional file 3: Fig S8c and Fig. 1d. The feature importances of the three selected STR analysis tools for Isaac- and BWA-aligned WGS are shown in Additional file 3: Figure S8d and Fig. 1e. Among the STR tools, TREDPARSE and STRetch ranked first in Isaac and BWA alignments, respectively. This model with the optimized parameters performed better than the default analysis, detecting all full-mutations in both Isaac and BWA test data.

### Analysis of known disease STRs in clinical NGS data

The decision rules that emerged from the modified analyses suggest the best approach to categorizing full-mutations in BWA data is to use STRetch, EH_v3, and exSTRa (see Fig. 1). Applying this pipeline on our BWA-aligned patient ES and WGS data, we identified 21 samples of interest, 10 with full-mutation expansions of the *AR*, *ATXN1*, *ATXN8*, *DMPK*, *FXN*, or *HTT* locus, two with borderline *ATXN2* alleles, and seven with intermediate or premutation alleles in the *FMR1* locus (summarized in Table 4). Additional file 2: Table S13 shows the EH_v3, STRetch, and exSTRa results of the identified STR candidates.

**Table 4:** Short tandem repeat candidates identified in our patient cohort

Sample ID	Gene	Inheritance	Sequencing	Pathogenic SNV/indel/SV finding	Phenotype	STR finding	Molecular validation
1901-P	*AR*	Inherited	WGS	No	Short stature, delayed gross motor, speech and language development, spasticity, cerebral palsy, and hypertonia	FM (full-penetrance)	FM (reduced/full-penetrance)
1901-F	*AR*	.	WGS	.	.	FM (full-penetrance)	FM (reduced/full-penetrance)
532-M	*ATXN1*	.	WGS	.	.	FM (full-penetrance)	n.a.
821-P	*ATXN2*	Inherited	ES	No	Mild intellectual disabilities, systemic hypertension, cutis aplasia, congenital heart defect, and limb anomalies	Borderline^a^	n.a.
821-M	*ATXN2*	.	ES	.	.	Borderline^a^	n.a.
1099-P	*ATXN8*	Inherited	ES	No	Hearing loss, cataract, myopia, visceral (kidney and spleen) cysts, proteinuria, and dysmorphic facial features	FM (higher penetrance)	n.a.
1099-M	*ATXN8*	.	ES	.	.	FM (higher penetrance)	n.a.
235-P	*ATXN8*	Inherited	WGS	No	Mild to moderate intellectual disability, and psychosis	FM (higher penetrance)	n.a.
235-M	*ATXN8*	.	WGS	.	.	FM (higher penetrance)	n.a.
2010-P	*DMPK*	Inherited	ES	Definite	Myotonic dystrophy type 1, inguinal hernias, joint hypermobility, strabismus, mild intellectual disability, and dysmorphic facial features	FM (full-penetrance)	FM (full-penetrance)
2010-M	*DMPK*	.	ES	.	Myotonic dystrophy type 1	FM (full-penetrance)	FM (full-penetrance)
148-M	*FMR1*	.	WGS	.	.	PM	n.a. (proband is negative for *FMR1* FM)
800-F	*FMR1*	.	WGS	.	.	IM	n.a.
480-P	*FMR1*	Inherited	WGS	Probable	Moderate intellectual disability, language delay, autism, borderline macrocephaly, low set ears, down slanting palpebral fissures, high palate, and soft skin	PM	n.a.
712-M	*FMR1*	.	WGS	.	.	PM	n.a. (proband is negative for *FMR1* FM)
925-P	*FMR1*	Inherited	WGS	No	Intellectual disability, developmental delay including speech delay, dysmorphic features, and behavioral challenges	PM	Negative for FM
925-S	*FMR1*	Inherited	WGS	No	Intellectual disability, autism, developmental delay, and dysmorphic features	IM	n.a.
925-M	*FMR1*	.	WGS	.	.	PM	n.a.
1987-F	*FXN*	.	WGS	.	.	NL/FM	Heterozygous NL/FM carrier
1530-P	*HTT*	Inherited	WGS	Uncertain	Global developmental delay, seizures, gliosis, developmental regression, encephalomalacia, hirsutism, nystagmus, optic atrophy, cyanosis, abnormal muscle tone, scoliosis, hearing impairment, and otitis media	FM (reduced penetrance)	FM (reduced penetrance)
1530-F	*HTT*	.	WGS	.	.	FM (reduced penetrance)	FM (reduced penetrance)

Probands with an identified STR candidate are given a “-P” suffix in the “Sample ID” column; sibling of the proband, “-S”; mother, “-M”; and father, “-F”. The genes harboring the STR candidate identified by our bioinformatics workflow and the inheritance pattern deciphered by comparing the proband’s STR call with that of the parents are reported. The “Sequencing” column shows the technology used: whole-genome sequencing (WGS) or exome sequencing (ES). The “Pathogenic SNV/indel/SV Finding” column indicates whether the proband has had a definite, probable, certain, or no diagnosis of a single-nucleotide variant (SNV), indel, or structural variant (SV). Phenotypic presentations reported in the probands, STR finding from our bioinformatics analysis, and the results from the molecular validation (if available) are also presented. *NL* normal, *IM* intermediate, *PM* premutation, *FM* full-mutation, *n.a.* not available

^a^Reduced-penetrance alleles have 33–34 repeats and full-penetrance alleles have ≥ 37 repeats

We found that all probands with an identified STR candidate inherited the allele from a parent (Additional file 2: Table S13). The inherited expansions either remained unchanged, decreased by one or a few repeat units, or increased by 1 to ~ 15 repeats during intergenerational transmission.

All individuals who tested negative in their clinical molecular assessments for *FMR1*, *FXN*, *SCA*, or *DMPK* full-mutation expansions were also categorized as non-expanded by our bioinformatics workflow (data not shown). In the ES data of a proband (2010-P) and his mother (2010-M) who had DM1 with *DMPK* full-mutation (> 50 repeats) findings upon prior molecular assessment, EH_v3 and exSTRa identified the full-mutation expansion. However, the repeat length estimated by EH_v3 in 2010-P and 2010-M was ~ 50 repeats, which is significantly lower than the molecular findings of 150 repeats in 2010-P and 430 repeats in 2010-M (Additional file 2: Table S13).

Based on the repeat lengths estimated by EH_v3, we categorized the identified full-mutations as reduced- or full-penetrance (Table 4; the different repeat size ranges associated with reduced- and full-penetrance of the STR expansion disorders are summarized in Additional file 2: Table S5). Six of the full-mutations we identified in the probands and parents were in the fully penetrant or higher penetrance repeat size range, while two were in the reduced-penetrance range.

We performed PCR-based molecular tests to verify the expansion status of a subset of the identified full-mutations (molecular findings summarized in the last column of Table 4 and Additional file 2: Table S13). The *HTT* full-mutations identified by EH_v3 (37 repeats), STRetch, and exSTRa in a proband (1530-P) and his father (1530-F) were concordant with the molecular results (37 ± 1 repeats). The *AR* full-mutations in a father (1901-F) and proband (1905-P) identified by EH_v3 (38 repeats) and STRetch were consistent with the PCR results (37 ± 1 repeats). In addition, the *FXN* candidate in a father (1987-F) identified by EH_v3 (69 repeats) was verified as full-mutation by PCR (87 ± 1 repeats). However, the clinical PCR test could not determine the exact repeat length of this allele due to the presence of sequence interruptions. In two samples (1099-P and 1099-M), EH_v3 did not return genotype calls for the *ATXN8* locus, which appears to be expanded as per STRetch and exSTRa. Modifying the *ATXN8* locus in the EH_v3 catalog to {“VariantType”: “Repeat”, “LocusId”: “ATXN8”, “LocusStructure”: “(CTG)*”, “ReferenceRegion”: “13:70713516-70713560”} helped with successful genotyping and identification of full-mutation with > 200 CTG repeats in both samples.

A *TBP* full-mutation identified by EH_v3 (54 repeats), but not supported by either STRetch or exSTRa, could not be verified by PCR (37 ± 1 repeats) (data not shown). Closer inspection of EH’s *TBP* genotype using GraphAlignmentViewer [31] revealed the lack of reads supporting the genotype call made in 1992-M. Visualization of candidate STRs, particularly those that were detected only by EH but not supported by either STRetch or exSTRa, may help reduce the number of false positives and confirmatory molecular tests required.

Lastly, we investigated the genotype calls of disease STRs made by EH_v2, EH_v3, and GangSTR in our patient ES and WGS datasets to see if the normal allele frequency distribution at these loci agreed with the reported population frequencies of normal alleles (Additional file 3: Fig S9 and S10, and Additional file 2: Table S14). In general, the repeat length distribution pattern of the STR alleles for most loci was consistent across the ES (Additional file 3: Fig S9) and WGS (Additional file 3: Fig S10) data, except for the *FMR1* and *FMR2* loci, which were characterized inconsistently in ES data. EH_v3 genotyped fewer *ATXN8* alleles and had a different repeat length distribution profile for the *ATXN7* and *HTT* loci in ES data. For the *CSTB* locus, more 1-repeat genotype calls were made by the tools in ES data, while we found none in WGS data.

More than half of the individuals in our clinical cohort are of European ancestry, so we compared the frequency of the three most common alleles ascertained in WGS data to the common normal allele in the Caucasian population reported in the literature (Additional file 2: Table S14). Except for a few loci, the repeat lengths of most common alleles determined by the tools were generally in good agreement with the reported repeat length of the common normal alleles in the Caucasian population.

## Discussion

The contribution of STR expansions to disease is just beginning to be understood. About 40 neurological disorders have been found to be caused by STR expansion mutations [2], with some recent studies reporting the identification of additional pathogenic STR expansion loci through NGS or the more advanced third-generation long-read sequencing technologies [32–36]. While repeat expansion disorders typically have a delayed age of onset, pre-symptomatic individuals have been reported to exhibit subtle cognitive, behavioral, or structural changes in the brain [37, 38]. Pre-symptomatic detection can help elucidate the pathophysiology and progression of the disease [39] and promote the development and timely implementation of disease-modifying interventions [40].

The challenges in detecting and characterizing repeat lengths of STR expansions in short-read NGS data are well recognized [41]. However, recent algorithmic improvements facilitate the detection of STR expansions that exceed read and/or fragment lengths, providing the opportunity to analyze a larger number of known disease STR loci simultaneously through ES or WGS [1, 10–14]. The analysis of STRs in clinical NGS data using these improved bioinformatics methods may help detect pathogenic full-mutation expansions and identify individuals with at-risk premutation, intermediate, or mutable normal alleles that may expand into an full-mutation in subsequent generations.

Of the available STR analysis tools, EH, GangSTR, and TREDPARSE are particularly valuable because they leverage evidence beyond reads that span an STR, enabling the genotyping of larger repeat expansions than is possible with spanning-read-only methods (lobSTR, HipSTR, and RepeatSeq). In this study, we have shown that EH, GangSTR, and TREDPARSE are also more reliable in genotyping STRs within read length compared to lobSTR, HipSTR, and RepeatSeq. STRetch and exSTRa can detect STR expansions but they do not reliably genotype them (STRetch) or do not genotype them at all (exSTRa). GangSTR and STRetch may also be useful in scanning the entire genome or exome for novel disease-causing STR expansions because they are not limited to the analysis of a prespecified catalog of known disease STRs, as other tools are.

We have shown through a systematic analysis that the choice of aligner could impact the performance of the STR analysis tools. The differences in the number of unmapped reads and reads with zero mapping quality may explain some of these performance inconsistencies we noted comparing the STR outputs from Isaac- and BWA-aligned data (Additional file 3: Fig S11). Selecting methods that function optimally with a given aligner appears to be a critical first step in designing a clinical bioinformatics workflow to screen STR expansions in NGS data.

In-line with earlier studies [10, 12], we found the characterization of *FMR1* full-mutations to be challenging. Homozygous *FXN* full-mutations were also refractory to detection because the genotypers typically identified only one of two full-mutation alleles. With our modified analysis, some of the tools detected more full-mutations at these loci. Notably, exSTRa performed better with an external control cohort, detecting all *FXN* and *FMR1* full-mutations in BWA data. Also, reducing the repeat length thresholds from full-mutation to intermediate size range enabled the detection of *FMR1* full-mutations with EH_v2, EH_v3, and TREDPARSE. With this reduced cut-off, the tools may also detect some intermediate and premutation carriers who, although are not affected, may be at risk of having children with fragile X syndrome if their intermediate/premutation allele is highly unstable and/or susceptible to late-onset conditions [42]. Early detection and genetic counseling of these at-risk individuals may, therefore, help intermediate/premutation allele carriers make informed reproductive decisions [42].

Our decision tree analyses favored using an ensemble approach that combined three of the most important STR methods (TREDPARSE, EH_v3, and STRetch for Isaac-aligned datasets, and STRetch, EH_v3, and exSTRa for BWA-aligned datasets). Comparing the best ensemble model with the stand-alone performance of the important tools revealed the advantage of adopting the ensemble approach (Additional file 2: Table S15). In BWA data, although exSTRa and EH_v3 alone had the same 100% recall as the ensemble model, the precision of individual tools was 4–24% lower compared to the ensemble model, showing that the latter has the potential to reduce or eliminate false positives while maintaining high sensitivity. This is consistent with our clinical cohort analysis wherein a full-mutation in *TBP* locus identified only by EH_v3 could not be verified by PCR; however, seven full-mutations identified by EH_v3 together with STRetch and/or exSTRa were confirmed by clinical PCR tests (Additional file 2: Table S13).

Our ensemble pipeline of EH_v3, STRetch, and exSTRa takes ~ 2.5 h per genome. Most methods, including EH_v3 and exSTRa, can perform targeted disease STR analysis on a single CPU within a matter of few seconds to minutes (Additional file 2: Table S16). STRetch took 2 h:24 min per genome on 16 CPUs as it profiles a larger catalog of STRs (~ 300 k) genome-wide and performs statistical analysis to detect outliers.

For *DMPK* assessment with EH (the default catalog file of which does not include OTS), we recommend including OTS as this results in a significant improvement in the repeat length estimation, particularly in WGS data. Although the threshold for defining pathogenic *DMPK* full-mutations that cause DM1 is only 50 repeats and EH and other tools detect *DMPK* full-mutations with 100% sensitivity, the different clinical forms of DM1 (mild, classic, and congenital), associated with varying severity and age of onset of symptoms are caused by *DMPK* full-mutations in the ranges of 50–~ 150, ~ 100–~ 1000, and > 1000 repeat units, respectively [43]. We show that with OTS, EH performs better at sizing *DMPK* full-mutations that ranged from ~ 130 to over 2000 repeats in the EGA WGS data and yields estimates that correlate better with full-mutation repeat lengths in these individuals (Additional file 3: Fig S12).

Although the methods we tested perform well in detecting and sizing full-mutations, for some disease STR loci, the difference between a non-full-mutation and a full-mutation, or between a reduced-penetrance and full-penetrance full-mutation is only a few repeat units, making it difficult to discriminate between these borderline alleles and their clinical significance. This limitation is also inherent to PCR-based tests as DNA polymerase slippage during STR amplification may result in under- or over-estimation of STR size by one or two repeat units [44].

Using our approach, we were able to confirm the presence of a clinically validated *DMPK* full-mutation in the ES data of a proband and his mother with DM1, inherited *HTT* and *AR* full-mutations in two families, and also the presence of an *FXN* normal/full-mutation in a father using clinical PCR and capillary electrophoresis. Importantly, none of the 67 individuals who previously had a negative clinical *FMR1*, *FXN*, *SCA*, or *DMPK* test result were falsely identified as “expanded” by our computational workflow.

Our analysis demonstrates that combining genotyping and statistical STR analysis tools yields optimal results. Of the currently available genotypers that can detect pathogenic expansions, only GangSTR works on STRs genome-wide. To identify expansions at novel loci, there is a need to expand the utility of existing genotypers or develop complementary genotyping approaches to interrogate candidates beyond the limited catalog of known disease STRs. The STR methods we used to analyze our clinical ES and WGS data were selected based on decision tree classification analysis on WGS. The main limitation with ES is its inconsistency in coverage and GC-bias, but we found that as long as there are sufficient reads mapping to the locus, the tools that work on WGS also perform reasonably well on ES data.

## Conclusions

Clinical NGS datasets are typically screened for pathogenic SNVs, small indels, and SVs to diagnose the underlying cause of genetic disorders. However, STRs are often not analyzed due to the lack of a reliable and well-optimized computational pipeline. We aimed to demonstrate the utility of STR analysis methods as a first-tier screen for pathogenic expansions in clinical NGS data and to promote the rapid integration of STR callers into routine clinical genomic analyses. Through a thorough evaluation of eight existing STR callers (lobSTR, HipSTR, RepeatSeq, TREDPARSE, EH, GangSTR, STRetch, and exSTRa), we have outlined important factors, such as the choice of sequence aligners and parameter tweaks of STR callers, which can improve the sensitivity to detect pathogenic expansions. Using decision tree classification, we illustrated the best aligner-specific pipeline to detect STR expansions with optimal sensitivity and specificity. To demonstrate the utility of this pipeline, we screened the ES and WGS data of 301 patient-parent trios/quads and identified samples with full-mutation, borderline, and intermediate/premutation alleles. Clinical PCR confirmation of a subset of identified full-mutation expansions showed absolute concordance between molecular and bioinformatics findings. Our results show that the incorporation of our recommended pipeline of tools to analyze clinical ES and WGS data can reliably identify pathogenic STR expansions, which could promote cascade testing in affected families and improve the diagnostics, treatment, and management of repeat expansion disorders.

## Supplementary Information

**Additional file 1: Table S1**: Experimental repeat size data of the samples in the Coriell European Genome-phenome Archive dataset. **Table S2**: Short tandem repeat alleles simulated in *C9orf72*, *FMR1*, and *FMR2* genes using ART simulator.

**Additional file 2: Table S3**: CAUSES exome sequence data summary. **Table S4**: Commands and parameters used for the implementation of lobSTR, RepeatSeq, HipSTR, TREDPARSE, ExpansionHunter, STRetch, exSTRa, and GangSTR. **Table S5**: Summary of repeat loci in catalogs used by short tandem repeat analysis methods. **Table S6**: Short tandem repeat calls of ExpansionHunter, GangSTR, TREDPARSE, lobSTR, HipSTR, RepeatSeq, STRetch, and exSTRa in the Isaac-aligned European Genome-phenome Archive and simulated genomes. **Table S7**: Short tandem repeat calls of ExpansionHunter, GangSTR, TREDPARSE, lobSTR, HipSTR, RepeatSeq, STRetch, and exSTRa in the BWA-aligned European Genome-phenome Archive and simulated genomes. **Table S8**: Known normal alleles characterised by ExpansionHunter, GangSTR, TREDPARSE, lobSTR, HipSTR, and RepeatSeq in the European Genome-phenome Archive and simulated genomes. **Table S9**: Known *FMR1* intermediate and premutation alleles characterised by ExpansionHunter, GangSTR, TREDPARSE, lobSTR, HipSTR, and RepeatSeq in the European Genome-phenome Archive and simulated genomes. **Table S10**: Full-mutation alleles characterised by ExpansionHunter, GangSTR, and TREDPARSE in the European Genome-phenome Archive and simulated genomes. **Table S11**: Performance of short tandem repeat callers on simulated and real genomes with *FMR1* CGG-repeat expansions in normal, premutation, and full-mutation size ranges. **Table S12**: Analysis and detection of *DMPK*, *FMR1*, and *FXN* full-mutation alleles using ExpansionHunter version 2 and GangSTR with and without off-target sites. **Table S13**: ExpansionHunter, STRetch, and exSTRa calls and molecular validation results of the short tandem repeat candidates identified in the CAUSES and IMAGINE genomes and exomes. **Table S14**: Repeat length distributions of the genotyped *AR*, *ATN1*, *ATXN1*, *ATXN2*, *ATXN3*, *ATXN7*, *ATXN8*/*ATXN8OS*, *ATXN10*, *C9orf72*, *CACNA1A*, *CBL*, *CNBP*, *CSTB*, *DMPK*, *FMR1*, *FMR2*, *FXN*, *HTT*, *JPH3*, *NOP56*, *PHOX2B*, *PPP2R2B*, and *TBP* alleles in the CAUSES and IMAGINE genomes. **Table S15**: Summary of performance metrics of the best model and individual STR tools in BWA modified analysis. **Table S16**: Run-time and peak memory requirements of short tandem repeat callers and aligners on a 40x human genome.

**Additional file 3: Fig S1**: exSTRa plots of EGA and simulated genomes. **Fig S2**: Decision tree model of the default analysis of Isaac-aligned EGA genomes on the training dataset. **Fig S3**: Performance metrics of the decision tree model in the default analysis of Isaac-aligned EGA genomes on the test dataset. **Fig S4**: Decision tree model of the default analysis of BWA-aligned EGA genomes on the training dataset. **Fig S5**: Performance metrics of the decision tree model in the default analysis of BWA-aligned EGA genomes on the test dataset. **Fig S6**: exSTRa plots of EGA genomes analyzed with 100 controls. **Fig S7**: Decision tree model of the modified analysis of Isaac-aligned EGA genomes on the training dataset. Fig S8: Performance metrics of decision tree model in the modified analysis of Isaac-aligned EGA test dataset. **Fig S9**: Allele frequency distribution of analyzed disease short tandem repeat loci in the CAUSES exomes. **Fig S10**: Allele frequency distribution of analyzed disease short tandem repeat loci in the CAUSES and IMAGINE genomes. **Fig S11**: Coverage and alignment statistics of Isaac- and BWA-aligned EGA genomes. **Fig S12**: Analysis of the *DMPK* locus by ExpansionHunter version 2 with and without off-target sites in the EGA dataset.

## Data Availability

The PCR-free whole-genome sequencing data of Coriell samples with known short tandem repeat expansions analyzed in the current study can be accessed in the European Genome-phenome Archive repository: https://www.ebi.ac.uk/ega/datasets/EGAD00001003562 [10]. Individual patient clinical or genomic data from the CAUSES [25] and IMAGINE [26] Studies are not available because they are potentially individually identifiable and not consented for public release. Investigators who wish access to the summary data of CAUSES and IMAGINE Studies can contact Dr. Jan Friedman (jmf@bcchr.ca). Results of all analyses based on the European Genome-phenome Archive, CAUSES, and IMAGINE datasets are included in this published article and its supplementary information files. lobSTR — https://github.com/mgymrek/lobstr-code RepeatSeq — https://github.com/adaptivegenome/repeatseq HipSTR — https://github.com/tfwillems/HipSTR TREDPARSE — https://github.com/humanlongevity/tredparse ExpansionHunter — https://github.com/Illumina/ExpansionHunter STRetch — https://github.com/Oshlack/STRetch exSTRa — https://bahlolab.github.io/exSTRa/doc/exSTRa.html GangSTR — https://github.com/gymreklab/GangSTR Ambry IDT bed — https://www.idtdna.com/pages/products/next-generation-sequencing/hybridization-capture/lockdown-panels/xgen-exome-research-panel-v2 Centogene bed — https://support.illumina.com/sequencing/sequencing_kits/nextera-rapid-capture-exome-kit/downloads.html GSC bed — https://earray.chem.agilent.com/suredesign/home.htm ART — https://www.niehs.nih.gov/research/resources/software/biostatistics/art/index.cfm BWA — http://bio-bwa.sourceforge.net/ Isaac — https://github.com/Illumina/Isaac4 scikit-learn — https://scikit-learn.org/stable/ MLxtend — http://rasbt.github.io/mlxtend/ European Genome-phenome Archive dataset — https://www.ebi.ac.uk/ega/datasets/EGAD00001003562 Picard — https://broadinstitute.github.io/picard/ CAUSES Study — https://bcchr.ca/news/causes-research-clinic-children-and-families-undiagnosed-disorders IMAGINE Study — https://www.child-bright.ca/imagine GraphAlignmentViewer — https://github.com/Illumina/GraphAlignmentViewer

## Abbreviations

*AR*: Androgen receptor
*ATN*: Atrophin
*ATXN*: Ataxin
AUC: Area under the curve
bp: Base pair
BWA: Burrows-Wheeler Aligner
*C9orf72*: Chromosome 9 open reading frame 72
CAUSES: Clinical Assessment of the Utility of Sequencing and Evaluation as a Service
DM: Myotonic dystrophy
*DMPK*: Dystrophia Myotonica Protein Kinase
EGA: European Genome-phenome Archive
EH: ExpansionHunter
ES: Exome sequencing
FM: Full-mutation
*FMR*: Fragile X mental retardation
FN: False negative
FP: False positive
*FXN*: Frataxin
*HTT*: Huntingtin
IM: Intermediate
IMAGINE: Integrated Metabolomics And Genomics In Neurodevelopment
IRR: In-repeat reads
NGS: Next-generation sequencing
NL: Normal
OTS: Off-target sites
PCR: Polymerase chain reaction
PM: Premutation
ROC: Receiver operating characteristic
SCA: Spinocerebellar ataxia
SNV: Single-nucleotide variant
STR: Short tandem repeat
*TBP*: TATA-box binding protein
TN: True negative
TP: True positive
WGS: Whole-genome sequencing
