# Correction to: Genome-wide sequencing as a first-tier screening test for short tandem repeat expansions

**DOI:** 10.1186/s13073-021-00961-4

**Published:** 2021-09-13

**Authors:** Indhu-Shree Rajan-Babu, Junran J. Peng, Readman Chiu, IMAGINE Study, Patricia Birch, Madeline Couse, Colleen Guimond, Anna Lehman, Jill Mwenifumbo, Clara van Karnebeek, Jan Friedman, CAUSES Study, Shelin Adam, Christele Du Souich, Alison Elliott, Anna Lehman, Jill Mwenifumbo, Tanya Nelson, Clara van Karnebeek, Rajan-Babu, Jan Friedman, Chenkai Li, Arezoo Mohajeri, Egor Dolzhenko, Michael A. Eberle, Inanc Birol, Jan M. Friedman

**Affiliations:** 1 Department of Medical Genetics, University of British Columbia and Children’s & Women’s Hospital, Vancouver, BC V6H3N1 Canada; 2 Department of Medical and Molecular Genetics, King’s College London, Strand, London, WC2R 2LS UK; 3 Canada’s Michael Smith Genome Sciences Centre, BC Cancer Agency, Vancouver, BC V5Z4S6 Canada; 4 Bioinformatics Graduate Program, University of British Columbia, Vancouver, BC V6T1Z4 Canada; 5 Illumina Inc., San Diego, CA 92121 USA

**Correction to: Genome Med 13, 126 (2021)**

**https://doi.org/10.1186/s13073-021-00932-9**

It was highlighted that in the original article [1] the list of the authors belonging in the IMAGINE and CAUSES Study were erroneously interchanged. The original article has been updated.

**Acknowledgements**

We would like to thank all the CAUSES and IMAGINE Study investigators. CAUSES Study investigators include Shelin Adam, Christele Du Souich, Alison Elliott, Anna Lehman, Jill Mwenifumbo, Tanya Nelson, Clara van Karnebeek, Rajan-Babu et al. Genome Medicine (2021) 13:126 Page 13 of 15 and Jan Friedman. The CAUSES Study is funded by Mining for Miracles, British Columbia Children’s Hospital Foundation, and Genome British Columbia. IMAGINE Study investigators include Patricia Birch, Madeline Couse, Colleen Guimond, Anna Lehman, Jill Mwenifumbo, Clara van Karnebeek, and Jan Friedman. We thank Compute Canada for the Research Allocation Competitions allocation, which facilitated our analysis of the IMAG INE and EGA genomes, and Julia Handra for coordinating the STR molecular testing of the clinical samples.

## References

[1] Rajan-Babu (2021). Genome-wide sequencing as a first-tier screening test for short tandem repeat expansions. Genome Med.

