# Supplementary material for: Genome-wide sequencing as a first-tier screening test for short tandem repeat expansions
Source: Genome Med. 2021 Aug 9;13:126. doi: 10.1186/s13073-021-00932-9 (PMC8351082; doi:10.1186/s13073-021-00932-9)
Supplement: Supplementary file 1 — Additional file 1: Table S1: Experimental repeat size data of the samples in the Coriell European Genome-phenome Archive dataset. Table S2: Short tandem repeat alleles simulated in C9orf72, FMR1, and FMR2 genes using ART simulator. [file 13073_2021_932_MOESM1_ESM.pdf]

Additional file 1

Coriell European Genome-phenome Archive Dataset True Repeat Size

The genotype status and repeat length information for most of the analyzed Coriell genomes have been previously characterized by PCR and/or Southern blot analysis. The repeat length information for some normal and full-mutation alleles was not available on the Coriell website (www.coriell.org). Therefore, we performed a literature search to retrieve this information from other studies that have determined repeat lengths in these cell lines using PCR and/or Southern blot techniques. We used the Coriell data together with repeat lengths retrieved from other studies (cited in Additional file 1: Table S1 below) to create a consensus repeat size catalog (last column of Additional file 1: Table S1). We relied on Coriell's repeat length information for *AR*, *ATN1*, *ATXN1*, *ATXN3*, and *FXN* reference samples that lacked experimental data.

In general, PCR and capillary electrophoresis-based methods can determine the repeat lengths of most short tandem repeat alleles with fewer than ~150 copies of a trinucleotide repeat, while Southern blot is recommended to resolve hyperexpanded full-mutation alleles such as those in the *DMPK*, *FXN,* and *FMR1* loci (1). Therefore, for *HTT* reference samples with expansions that only ranged from 44 to 72 CAGs, we used data from a PCR study (2). For *DMPK* and *FMR1* reference samples, we relied on PCR-based estimates for most alleles in the normal to premutation size ranges and used available SB data for full-mutation expansions. The repeat size data for some normal *DMPK* and *FXN* alleles and *FMR1* full-mutation lengths of some fragile X samples could not be identified. Also, we could not establish the consensus size estimate for two samples (NA06896 and NA20241) which appear to be "mosaic" and were reported to have very varied repeat lengths by different studies. For a few other cell lines that showed two

distinct repeat lengths (e.g., NA06075 and NA09145) or had a slight variation in reported repeat lengths (e.g., NA20233 and NA20240), we used their mean values as consensus size.

Table S1: Experimental repeat size data of the samples in the Coriell European Genome-phenome Archive dataset.

Sample ID	Disease	Gene	Sex	Repeat motif	Coriell size	Genotype	Repeat sizes reported by other studies	Consensus repeat length
NA06075*	DM1	*DMPK*	Male	$(CTG)_n$	NL/66	NL/FM	12/56,70 (±0.9)(3)<br>12/55,71(4)<br>12/56,71(5)	12/56,71 [64 (mean)]
NA04567	DM1	*DMPK*	Female	$(CTG)_n$	NL/~700	NL/FM	21/637 (±33)(3)<br>21/>180(4)<br>21/>200(5)	21/637
NA05164	DM1	*DMPK*	Female	$(CTG)_n$	21/~340	NL/FM	21/377 (±53)(3)<br>21/>180(4)<br>21/>200(5)	21/377
NA04648	DM1	*DMPK*	Male	$(CTG)_n$	NL/~1000 (minor species at ~700-800)	NL/FM	5/1008 (±49)(3)<br>5/>180(4)<br>5/>200(5)	5/1008
NA05152	DM1	*DMPK*	Male	$(CTG)_n$	NL/~1500	NL/FM	5/1621 (±30)(3)<br>5/>200(5)	5/1621
NA23378	DM1	*DMPK*	Male	$(CTG)_n$	NL/~80-90	NL/FM	22/138 (±5)(3)<br>22/129-132(4)<br>22/75,126(5)	22/138
NA23374	DM1	*DMPK*	Female	$(CTG)_n$	NL/~130-140	NL/FM	NL/475(3)	NL/475
NA23300*	DM1	*DMPK*	Male	$(CTG)_n$	NL/~150-160	NL/FM	NL/550(3)<br>5/>180(4)<br>5/>200(5)	5/550
NA03986*	DM1	*DMPK*	Male	$(CTG)_n$	NL/<500	NL/FM	12/550*(3, 6)	12/550
NA03989	DM1	*DMPK*	Male	$(CTG)_n$	NL/<2000	NL/FM	13/>180(4)	13/2000
NA03990	DM1	*DMPK*	Female	$(CTG)_n$	NL/50-80	NL/FM	NL/78(3) (NA03990 - LCL);	14/78

							14/108-110(5) (NA03991-fibroblast)	
NA03696	DM1	*DMPK*	Female	$(CTG)_n$	NL/<1000	NL/FM	12/697 (±13)(3)<br>12/>165,>180(4)<br>12/>200(5)	12/697
NA03759	DM1	*DMPK*	Male	$(CTG)_n$	NL/<2000	NL/FM	14/>200(5)	14/2000
NA04034	DM1	*DMPK*	Male	$(CTG)_n$	NL/<1000	NL/FM	12/700(3, 6)	12/700
NA03697	DM1	*DMPK*	Male	$(CTG)_n$	NL/<500	NL/FM	12/412 (±33)(3)<br>12/>200(5)	12/412
NA03132	DM1	*DMPK*	Male	$(CTG)_n$	5/~1700	NL/FM	5/2078 (±217)(3)<br>5/>200(5)	5/2078
NA03756*	DM1	*DMPK*	Male	$(CTG)_n$	NL/<500	NL/FM	NL/450(3)<br>13/>200(5)	13/450
NA13716	DRPLA	*ATN1*	Male	$(CAG)_n$	16/68	NL/FM	Nil	16/68
NA13717	DRPLA	*ATN1*	Male	$(CAG)_n$	15/65	NL/FM	Nil	15/65
NA03816	FRDA	*FXN*	Female	$(GAA)_n$	~330/~380	FM/FM	Nil	330/380
NA04079	FRDA	*FXN*	Male	$(GAA)_n$	~420/~541	FM/FM	Nil	420/541
NA14519	FRDA	*FXN*	Female	$(GAA)_n$	9/~1335	NL/FM	Nil	9/1335
NA15850	FRDA	*FXN*	Male	$(GAA)_n$	~650/~1030	FM/FM	Nil	650/1030
NA15847	FRDA	*FXN*	Female	$(GAA)_n$	NL/760	NL/FM	Nil	NL/760
NA15848	FRDA	*FXN*	Male	$(GAA)_n$	NL/830	NL/FM	Nil	NL/830
NA16197	FRDA	*FXN*	Male	$(GAA)_n$	~760/~830	FM/FM	Nil	760/830
NA16200	FRDA	*FXN*	Female	$(GAA)_n$	NL/830	NL/FM	Nil	NL/830
NA16202	FRDA	*FXN*	Female	$(GAA)_n$	NL/830	NL/FM	Nil	NL/830
NA16203	FRDA	*FXN*	Female	$(GAA)_n$	~670/~830	FM/FM	Nil	670/830
NA16205	FRDA	*FXN*	Male	$(GAA)_n$	~530/~530	FM/FM	Nil	530/530
NA16209	FRDA	*FXN*	Female	$(GAA)_n$	~800/~800	FM/FM	Nil	800/800
NA16210	FRDA	*FXN*	Male	$(GAA)_n$	~580/~580	FM/FM	Nil	580/580
NA16212	FRDA	*FXN*	Female	$(GAA)_n$	NL/500	NL/FM	Nil	NL/500
NA16216	FRDA	*FXN*	Female	$(GAA)_n$	~200/~500	FM/FM	Nil	200/500

NA16213	FRDA	*FXN*	Male	$(GAA)_n$	NL/420	NL/FM	Nil	NL/420
NA16215	FRDA	*FXN*	Female	$(GAA)_n$	NL/830	NL/FM	Nil	NL/830
NA16214	FRDA	*FXN*	Male	$(GAA)_n$	~600/~700	FM/FM	Nil	600/700
NA16227	FRDA	*FXN*	Female	$(GAA)_n$	~630/~830	FM/FM	Nil	630/830
NA16229	FRDA	*FXN*	Female	$(GAA)_n$	NL/670	NL/FM	Nil	NL/670
NA16228	FRDA	*FXN*	Female	$(GAA)_n$	~670/~830	FM/FM	Nil	670/830
NA16237	FRDA	*FXN*	Female	$(GAA)_n$	NL/700	NL/FM	Nil	NL/700
NA16243	FRDA	*FXN*	Male	$(GAA)_n$	~670/~1170	FM/FM	Nil	670/1170
NA16240	FRDA	*FXN*	Male	$(GAA)_n$	NL/830	NL/FM	Nil	NL/830
NA16207	FRDA	*FXN*	Female	$(GAA)_n$	~280/~830	FM/FM	Nil	280/830
NA06895	FXS	*FMR1*	Male	$(CGG)_n$	23	NL	Nil	23
NA04025	FXS	*FMR1*	Male	$(CGG)_n$	645	FM	795 (SB); >250 (PCR)(7) >200(8, 9)	795
NA04926	FXS	*FMR1*	Male	$(CGG)_n$	Not reported	FM	Nil	-
NA05131	FXS	*FMR1*	Male	$(CGG)_n$	Not reported	FM	Nil	-
NA05185	FXS	*FMR1*	Male	$(CGG)_n$	Not reported	FM	Nil	-
NA09145	FXS	*FMR1*	Male	$(CGG)_n$	Not reported	FM	~660-990 (SB); >250 (PCR)(7) >200(8)	660-990 [825 (mean)]
NA09237	FXS	*FMR1*	Male	$(CGG)_n$	931-940	FM	1062 (SB); >250 (PCR)(7) >200(9)	1062
NA07063	FXS	*FMR1*	Female	$(CGG)_n$	Not reported	NL/FM	32/>200(10)	32/-
NA07539	FXS	*FMR1*	Male	$(CGG)_n$	23	NL	Nil	23
NA06890	FXS	*FMR1*	Male	$(CGG)_n$	30	NL	30(8-11)	30
NA06905	FXS	*FMR1*	Female	$(CGG)_n$	23/70	NL/PM	Nil	23/70
NA07536	FXS	*FMR1*	Male	$(CGG)_n$	23	NL	Nil	23
NA07540	FXS	*FMR1*	Female	$(CGG)_n$	23/29	NL/NL	Nil	23/29

NA07542	FXS	*FMR1*	Male	$(CGG)_n$	23	NL	Nil	23
NA06910	FXS	*FMR1*	Female	$(CGG)_n$	30/75-89	NL/PM	30/88(12)	30/88
NA06894	FXS	*FMR1*	Female	$(CGG)_n$	30/78	NL/PM	Nil	30/78
NA07541	FXS	*FMR1*	Female	$(CGG)_n$	29/31	NL/NL	29/31(8)	29/31
NA07175	FXS	*FMR1*	Female	$(CGG)_n$	23/30	NL/NL	23/30(10, 11)	23/30
NA06889	FXS	*FMR1*	Female	$(CGG)_n$	23/30	NL/NL	Nil	23/30
NA06893	FXS	*FMR1*	Female	$(CGG)_n$	23/30	NL/NL	Nil	23/30
NA06896*	FXS	*FMR1*	Female	$(CGG)_n$	23/95-120-140	NL/PM	23/115(10, 11)<br>NL/148-201 (SB);<br>23/112,136,153,175,>250(PCR)(7)<br>23/113,133-138,155,175,198,>200(8)<br>23/183(9)	23/No consensus
NA07538	FXS	*FMR1*	Female	$(CGG)_n$	29/29	NL/NL	29/29(8-11, 13, 14)	29/29
NA07537	FXS	*FMR1*	Female	$(CGG)_n$	28-29/>200	NL/FM	29/>200(8-11)<br>NL/329 (SB); 29/>250(PCR)(7)	29/329
NA06897	FXS	*FMR1*	Male	$(CGG)_n$	477	FM	>200(8, 9)	477
NA07174	FXS	*FMR1*	Male	$(CGG)_n$	30	NL	30(10)	30
NA06903	FXS	*FMR1*	Female	$(CGG)_n$	23/95	NL/PM	Nil	23/95
NA07543	FXS	*FMR1*	Female	$(CGG)_n$	20/29	NL/NL	Nil	20/29
NA06852	FXS	*FMR1*	Male	$(CGG)_n$	>200	FM	395 (SB); >250 (PCR)(7)<br>>200(8-11)	395
NA06891	FXS	*FMR1*	Male	$(CGG)_n$	~118	PM	~160(10, 11)[#]<br>119(8)<br>121(9)	119
NA06907	FXS	*FMR1*	Female	$(CGG)_n$	29/85	NL/PM	29/91(10, 11)	29/91
NA06906	FXS	*FMR1*	Male	$(CGG)_n$	96	PM	101(9)	101
NA06892	FXS	*FMR1*	Male	$(CGG)_n$	93	PM	93(9-11)<br>~110 (SB); 90 (PCR)(7)	93
NA06904	FXS	*FMR1*	Female	$(CGG)_n$	23/29	NL/NL	Nil	23/29

NA06968	FXS	*FMR1*	Female	$(CGG)_n$	32/107	NL/PM	Nil	32/107
NA07294	FXS	*FMR1*	Male	$(CGG)_n$	Not reported	FM	>200(10, 11)	-
NA09316	FXS	*FMR1*	Male	$(CGG)_n$	Not reported	FM	Nil	-
NA09317	FXS	*FMR1*	Male	$(CGG)_n$	Not reported	FM	Nil	-
NA09497	FXS	*FMR1*	Male	$(CGG)_n$	Not reported	FM	Nil	-
NA07730	FXS	*FMR1*	Male	$(CGG)_n$	Not reported	FM	Nil	-
NA03200	FXS	*FMR1*	Male	$(CGG)_n$	Not reported	FM	Nil	-
NA20235	FXS	*FMR1*	Female	$(CGG)_n$	29/45	NL/IM	29/45(8-11, 13, 14)<br>30/45(14)	29/45
NA20238	FXS	*FMR1*	Female	$(CGG)_n$	29/30	NL/NL	29/30(8, 10, 11, 13, 14)	29/30
NA20237	FXS	*FMR1*	Male	$(CGG)_n$	100-104	PM	139(9)<br>100,137(14)<br>99,135(14)	100,137 [119 (mean)]
NA20239	FXS	*FMR1*	Female	$(CGG)_n$	20/183-193	NL/FM	20/~200(8-11)<br>20/No consensus(13)<br>21/200(14)<br>21/202(14)	20/200
NA20242	FXS	*FMR1*	Female	$(CGG)_n$	30/73	NL/PM	30/74(10, 11, 14)<br>30/73,105(8)<br>30/105(9)<br>30/73(13, 14)	30/73
NA20243	FXS	*FMR1*	Female	$(CGG)_n$	29/41	NL/NL	29/41(8-11, 13, 14)	29/41
NA20230	FXS	*FMR1*	Male	$(CGG)_n$	53	IM	54(9-11, 14)<br>53(13)	54

NA20232	FXS	*FMR1*	Male	$(CGG)_n$	46	IM	46(9-11, 13, 14)	46
NA20233	FXS	*FMR1*	Male	$(CGG)_n$	117	PM	120(9)<br>117(13)<br>119(14)<br>118(14)	118 (mean)
NA20234	FXS	*FMR1*	Female	$(CGG)_n$	31/46	NL/IM	31/46(10, 11, 13, 14)<br>26/46(9)	31/46
NA20236	FXS	*FMR1*	Female	$(CGG)_n$	31/53	NL/IM	31/54(10, 11, 14)<br>31/53(13)	31/54
NA20231	FXS	*FMR1*	Male	$(CGG)_n$	76	PM	78(9-11, 14)<br>76(13)<br>77(14)	78
NA20240	FXS	*FMR1*	Female	$(CGG)_n$	30/80	NL/PM	30/87(10, 11)<br>30/81(8)<br>30/83(9)<br>30/80(13)<br>31/82(14)<br>31/81(14)	30/83 (mean)
NA20241	FXS	*FMR1*	Female	$(CGG)_n$	29/93-110	NL/PM	NL/103-130 (SB); 29/88,111,116 (PCR)(7)<br>29/125(9)<br>29/No consensus(13)<br>30/91(14)<br>29/90(14)	29/No consensus
NA20244	FXS	*FMR1*	Male	$(CGG)_n$	41	NL	41(9-11, 13, 14)	41
NA07862	FXS	*FMR1*	Male	$(CGG)_n$	501-550	FM	>200(8-11)	501-550 [525 (mean)]
NA13509	HD	*HTT*	Female	$(CAG)_n$	15/70	NL/FM	15/72(2)	15/72
NA13515	HD	*HTT*	Male	$(CAG)_n$	16/66	NL/FM	16/65(2)	16/65
NA13507	HD	*HTT*	Male	$(CAG)_n$	15/55	NL/FM	15/54(2)	15/54

NA13508	HD	*HTT*	Male	$(CAG)_n$	22/58	NL/FM	22/57(2)	22/57
NA13510	HD	*HTT*	Male	$(CAG)_n$	15/44	NL/FM	15/44(2)	15/44
NA13511	HD	*HTT*	Male	$(CAG)_n$	45/47	FM/FM	45/47(2)	45/47
NA13512	HD	*HTT*	Female	$(CAG)_n$	16/44	NL/FM	16/44(2)	16/44
NA13513	HD	*HTT*	Female	$(CAG)_n$	15/49	NL/FM	15/49(2)	15/49
NA13514	HD	*HTT*	Female	$(CAG)_n$	15/52	NL/FM	15/52(2)	15/52
NA13503	HD	*HTT*	Female	$(CAG)_n$	17/45	NL/FM	17/45(2)	17/45
NA13504	HD	*HTT*	Male	$(CAG)_n$	16/46	NL/FM	16/46(2)	16/46
NA13505	HD	*HTT*	Male	$(CAG)_n$	22/50	NL/FM	22/50(2)	22/50
NA13506	HD	*HTT*	Male	$(CAG)_n$	17/48	NL/FM	17/48(2)	17/48
NA06926	SCA1	*ATXN1*	Male	$(CAG)_n$	29/52	NL/FM	Nil	29/52
NA13536	SCA1	*ATXN1*	Female	$(CAG)_n$	31/43	NL/FM	Nil	31/43
NA13537	SCA1	*ATXN1*	Male	$(CAG)_n$	32/60	NL/FM	Nil	32/60
NA06151	SCA3	*ATXN3*	Male	$(CAG)_n$	24/74	NL/FM	Nil	24/74
NA23709	SBMA	*AR*	Male	$(CAG)_n$	51	FM	Nil	51

DM1: Myotonic Dystrophy Type 1; DRPLA: Dentatorubral-pallidoluysian atrophy; FRDA: Friedreich Ataxia; FXS: Fragile X Syndrome; HD: Huntington Disease; SCA1: Spinocerebellar Ataxia Type 1; SCA3: Spinocerebellar Ataxia Type 3; SMBA: Spinal and bulbar muscular atrophy

DMPK: Dystrophia Myotonica Protein Kinase; *ATN1*: Atrophin 1; *FXN*: Frataxin; *FMR1*: Fragile X Mental Retardation 1; *ATXN1*: Ataxin 1; *ATXN3*: Ataxin 3; *AR*: Androgen Receptor

NL: Normal; IM: Intermediate; PM: Premutation; FM: Full-mutation

*Samples that exhibit mosaicism

SB: Southern blot analysis; LCL: Lymphoblastoid Cell Line; PCR: Polymerase Chain Reaction

[#]Unstable expansion of a 118-repeat PM during cell culture

ART WGS Data Simulation

We simulated genomes using the ART next-generation sequencing read simulator (15). Expanded alleles at short tandem repeat loci in three genes were simulated from the hg19 human reference genome using the GATK FastaAlternateReferenceMaker tool (see Table S2 below). Mutated genomes were simulated at 15x coverage with 150 bp paired-end reads to yield R1 and R2 fastq files. An unmutated reference genome was also simulated with the same parameters. The following ART command was used to simulate the genomes using the built-in quality profile for the Illumina HiSeq X PCR-free sequencing system:

```
art_illumina -ss HSXn -i $fasta_file -l 150 -f 15 -o $output_prefix -m 500 -s 50
```

The simulated fastq files were aligned to the hg19 human reference genome using BWA.

Table S2: Short tandem repeat alleles simulated in *C9orf72, FMR1,* and *FMR2* genes using ART simulator.

**Gene**	**Associated Repeat Expansion Disorder**	**Allele Size (# of repeats)**
C9orf72	Amyotrophic lateral sclerosis	60, 500, 1000
FMR1	Fragile X syndrome	23, 29, 30, 70, 88, 93, 107, 200, 329, 477, 525, 795, 1062
FMR2	Fragile XE syndrome	200, 500, 1000

C9orf72: Chromosome 9 open reading frame 72
FMR1: fragile X mental retardation 1
FMR2: fragile X mental retardation 2

References

1. Filipovic-Sadic S, Sah S, Chen L, Krosting J, Sekinger E, Zhang W, et al. A novel FMR1 PCR method for the routine detection of low abundance expanded alleles and full mutations in fragile X syndrome. Clin Chem. 2010;56(3):399-408.
2. Thibert RJ, Nicholson K, Wisotsky J, Le V, Latham JG, Statt S. A Robust PCR/CE Assay Using AmplideX® Technology for Rapid and Accurate Genotyping of CAG Repeat Expansions in HTT. ACMG; Charlotte, North Carolina)2018.
3. Kalman L, Tarleton J, Hitch M, Hegde M, Hjelm N, Berry-Kravis E, et al. Development of a genomic DNA reference material panel for myotonic dystrophy type 1 (DM1) genetic testing. J Mol Diagn. 2013;15(4):518-25.
4. Lian M, Rajan-Babu IS, Singh K, Lee CG, Law HY, Chong SS. Efficient and highly sensitive screen for myotonic dystrophy type 1 using a one-step triplet-primed PCR and melting curve assay. J Mol Diagn. 2015;17(2):128-35.
5. Hall B, Kemppainen J, Wisotsky J, Culp K, Latham G, Bram E. P10.40D - Development of a streamlined molecular assay that determines both allele and expanded repeat size in D*MPK* for myotonic dystrophy 1. The European Society of Human Genetics; Copenhagen, Denmark)2017.
6. Hall B, Snyder PJ, Kemppainen J, Prior TW, Rao PR, Latham GJ. Clinical correlations of a streamlined molecular assay based on AmplideX® PCR/CE technology that determines repeat size for both normal and expanded alleles in DMPK for myotonic dystrophy type 1. ASHG 20172017.
7. Chen L, Hadd A, Sah S, Houghton JF, Filipovic-Sadic S, Zhang W, et al. High-resolution methylation polymerase chain reaction for fragile X analysis: evidence for novel FMR1 methylation patterns undetected in Southern blot analyses. Genet Med. 2011;13(6):528-38.
8. Chen L, Hadd A, Sah S, Filipovic-Sadic S, Krosting J, Sekinger E, et al. An information-rich CGG repeat primed PCR that detects the full range of fragile X expanded alleles and minimizes the need for southern blot analysis. J Mol Diagn. 2010;12(5):589-600.
9. Lim GX, Yeo M, Koh YY, Winarni TI, Rajan-Babu IS, Chong SS, et al. Validation of a commercially available test that enables the quantification of the numbers of CGG trinucleotide repeat expansion in FMR1 gene. PLoS One. 2017;12(3):e0173279.
10. Rajan-Babu IS, Law HY, Yoon CS, Lee CG, Chong SS. Simplified strategy for rapid first-line screening of fragile X syndrome: closed-tube triplet-primed PCR and amplicon melt peak analysis. Expert Rev Mol Med. 2015;17:e7.
11. Rajan-Babu IS, Teo CR, Lian M, Lee CG, Law HY, Chong SS. Single-tube methylation-specific duplex-PCR assay for rapid and accurate diagnosis of Fragile X Mental Retardation 1-related disorders. Expert Rev Mol Diagn. 2015;15(3):431-41.
12. Lyon E, Laver T, Yu P, Jama M, Young K, Zoccoli M, et al. A simple, high-throughput assay for Fragile X expanded alleles using triple repeat primed PCR and capillary electrophoresis. J Mol Diagn. 2010;12(4):505-11.
13. Amos Wilson J, Pratt VM, Phansalkar A, Muralidharan K, Highsmith WE, Beck JC, et al. Consensus characterization of 16 FMR1 reference materials: a consortium study. J Mol Diagn. 2008;10(1):2-12.
14. Juusola JS, Anderson P, Sabato F, Wilkinson DS, Pandya A, Ferreira-Gonzalez A. Performance evaluation of two methods using commercially available reagents for PCR-based detection of FMR1 mutation. J Mol Diagn. 2012;14(5):476-86.

15. Huang W, Li L, Myers JR, Marth GT. ART: a next-generation sequencing read simulator. Bioinformatics. 2012;28(4):593-4.
