# Supplementary material for: Genome-wide sequencing as a first-tier screening test for short tandem repeat expansions
Source: Genome Med. 2021 Aug 9;13:126. doi: 10.1186/s13073-021-00932-9 (PMC8351082; doi:10.1186/s13073-021-00932-9)

Additional file 3

Fig S1: exSTRa plots of EGA and simulated genomes. a. Empirical distribution function (ECDF; left) and t-sum (right) plots of Isaac-aligned data. In the ECDF plots, samples with a known expansion at the tested STR locus were colored red and the rest of the samples were colored black. The t-sum plots show the samples identified by exSTRa to harbor a significant expansion at the given locus. The title on top of each plot indicates the STR locus, the number of repeats in the hg19 reference genome, and the pathogenic lower bound number of repeats of the STR. **b.** ECDF (left) and t-sum (right) plots of BWA-aligned data.

Fig S1

a

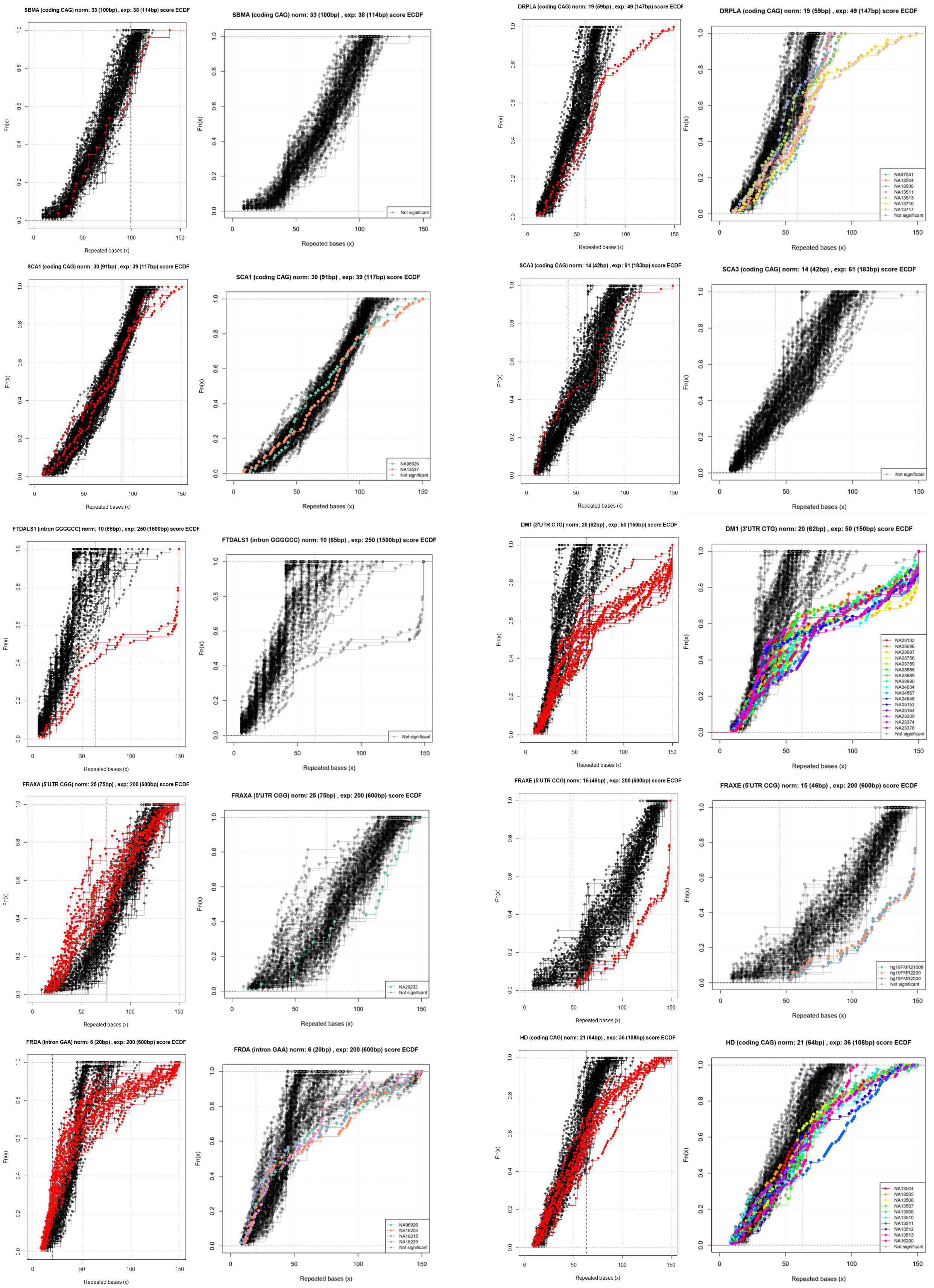

SBMA (coding CAG) norm: 33 (100bp) , exp: 38 (114bp) score ECDF
Not significant
DRPLA (coding CAG) norm: 19 (59bp) , exp: 49 (147bp) score ECDF
NA07541
NA13504
NA13506
NA13511
NA13513
NA13716
NA13717
Not significant
SCA1 (coding CAG) norm: 30 (91bp) , exp: 39 (117bp) score ECDF
NA06926
NA13537
Not significant
SCA3 (coding CAG) norm: 14 (42bp) , exp: 61 (183bp) score ECDF
Not significant
FTDALS1 (intron GGGGCC) norm: 10 (65bp) , exp: 250 (1500bp) score ECDF
Not significant
DM1 (3'UTR CTG) norm: 20 (62bp) , exp: 50 (150bp) score ECDF
NA03132
NA03696
NA03697
NA03756
NA03759
NA03986
NA03989
NA03990
NA04034
NA04567
NA04648
NA05152
NA05164
NA23300
NA23374
NA23378
Not significant
FRAXA (5'UTR CGG) norm: 25 (75bp) , exp: 200 (600bp) score ECDF
NA20232
Not significant
FRAXE (5'UTR CCG) norm: 15 (46bp) , exp: 200 (600bp) score ECDF
hg19FMR21000
hg19FMR2200
hg19FMR2500
Not significant
FRDA (intron GAA) norm: 6 (20bp) , exp: 200 (600bp) score ECDF
NA06926
NA16205
NA16215
NA16229
Not significant
HD (coding CAG) norm: 21 (64bp) , exp: 36 (108bp) score ECDF
NA13504
NA13505
NA13506
NA13507
NA13508
NA13510
NA13511
NA13512
NA13513
NA16200
Not significant
Fn(x)
Repeated bases (x)

Fig S1

b

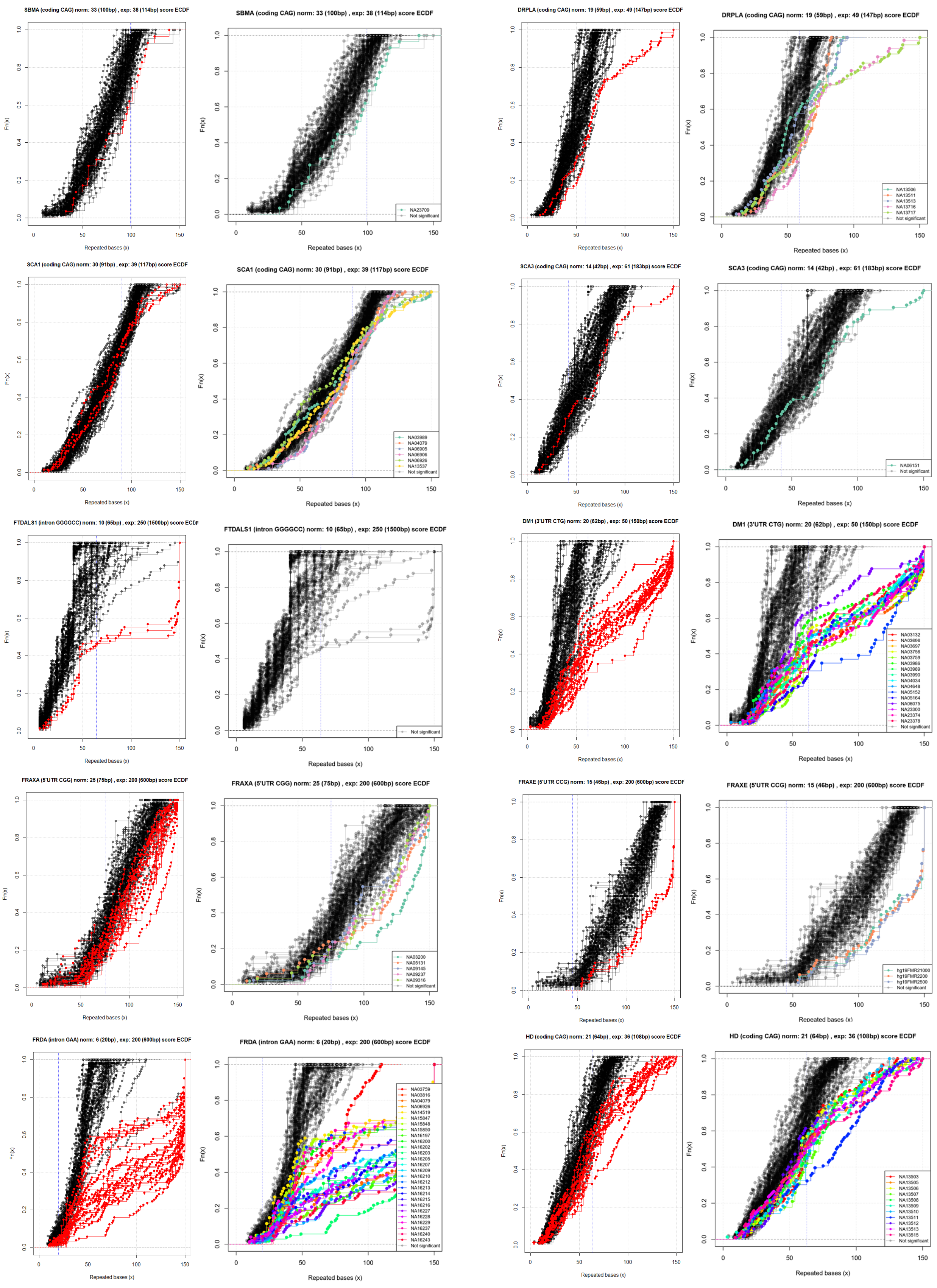

SBMA (coding CAG) norm: 33 (100bp) , exp: 38 (114bp) score ECDF
NA23709
Not significant
DRPLA (coding CAG) norm: 19 (59bp) , exp: 49 (147bp) score ECDF
NA13506
NA13511
NA13513
NA13716
NA13717
Not significant
SCA1 (coding CAG) norm: 30 (91bp) , exp: 39 (117bp) score ECDF
NA03989
NA04079
NA06905
NA06906
NA06926
NA13537
Not significant
SCA3 (coding CAG) norm: 14 (42bp) , exp: 61 (183bp) score ECDF
NA06151
Not significant
FTDALS1 (intron GGGGCC) norm: 10 (65bp) , exp: 250 (1500bp) score ECDF
Not significant
DM1 (3'UTR CTG) norm: 20 (62bp) , exp: 50 (150bp) score ECDF
FRAXA (5'UTR CGG) norm: 25 (75bp) , exp: 200 (600bp) score ECDF
NA03200
NA05131
NA09145
NA09237
NA09316
Not significant
FRAXE (5'UTR CCG) norm: 15 (46bp) , exp: 200 (600bp) score ECDF
hg19FMR21000
hg19FMR2200
hg19FMR2500
Not significant
FRDA (intron GAA) norm: 6 (20bp) , exp: 200 (600bp) score ECDF
HD (coding CAG) norm: 21 (64bp) , exp: 36 (108bp) score ECDF
Fn(x)
Repeated bases (x)

Fig S2: Decision tree model of the default analysis of Isaac-aligned EGA genomes on the training dataset. Decision tree generated by the classifier on the training dataset (n=942). Node #0 at the top of the tree is the root node. Each node lists an STR tool (feature). The "samples" number represents the total number of data points present within a particular node, and "value" shows the number of expanded (full-mutation, FM) and non expanded (non-FM) data points. The shade of the colour of each node reflects the proportion of expanded to non-expanded data points, with deeper orange and blue meaning more non expanded and expanded data points, respectively. Gini index shows the impurity at each node. The terminal nodes or leaves with a Gini value of 0 have data points belonging entirely to either the expanded or the non-expanded class. EHv2: ExpansionHunter version 2; Expanded and Not_Expanded imply the presence and absence of FM, respectively.

Fig S2

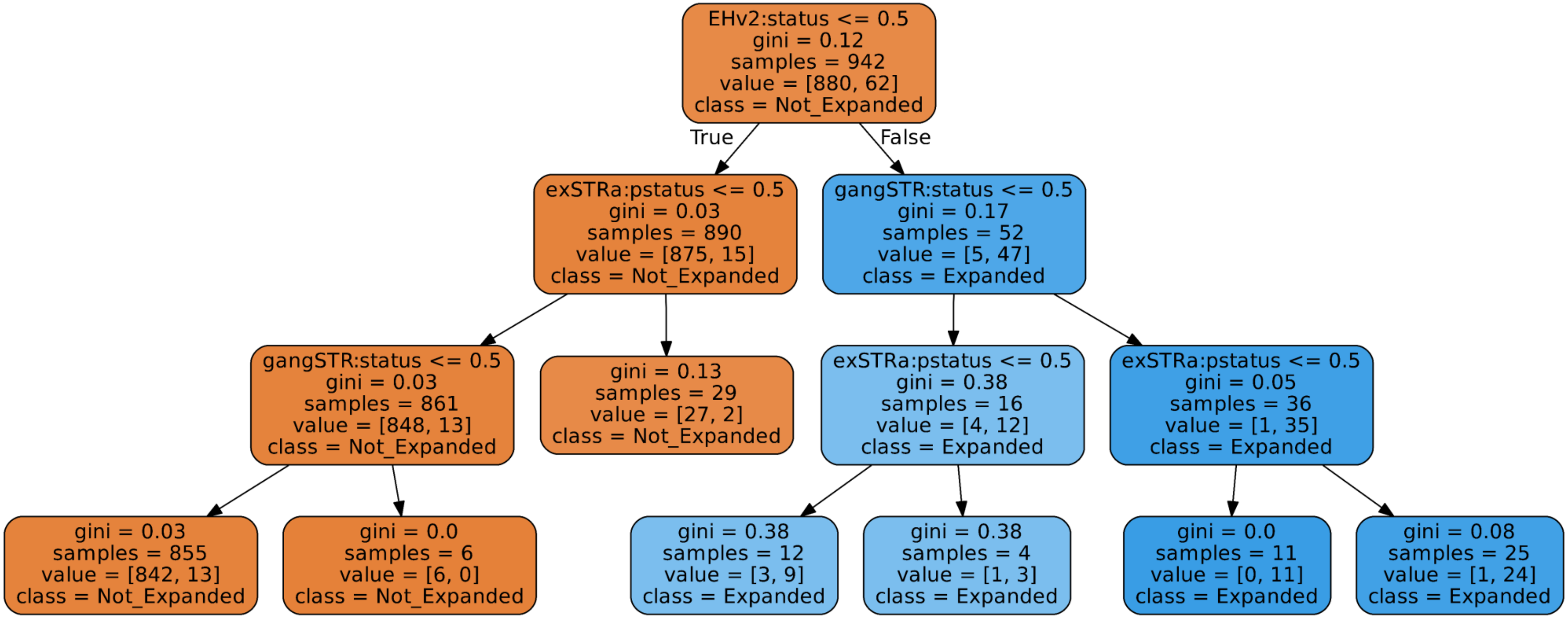
EHv2:status <= 0.5
gini = 0.12
samples = 942
value = [880, 62]
class = Not_Expanded
True
False
exSTRa:pstatus <= 0.5
gini = 0.03
samples = 890
value = [875, 15]
class = Not_Expanded
gangSTR:status <= 0.5
gini = 0.17
samples = 52
value = [5, 47]
class = Expanded
gangSTR:status <= 0.5
gini = 0.03
samples = 861
value = [848, 13]
class = Not_Expanded
gini = 0.13
samples = 29
value = [27, 2]
class = Not_Expanded
exSTRa:pstatus <= 0.5
gini = 0.38
samples = 16
value = [4, 12]
class = Expanded
exSTRa:pstatus <= 0.5
gini = 0.05
samples = 36
value = [1, 35]
class = Expanded
gini = 0.03
samples = 855
value = [842, 13]
class = Not_Expanded
gini = 0.0
samples = 6
value = [6, 0]
class = Not_Expanded
gini = 0.38
samples = 12
value = [3, 9]
class = Expanded
gini = 0.38
samples = 4
value = [1, 3]
class = Expanded
gini = 0.0
samples = 11
value = [0, 11]
class = Expanded
gini = 0.08
samples = 25
value = [1, 24]
class = Expanded

Fig S3: Performance metrics of the decision tree model in the default analysis of Isaac-aligned EGA genomes on the test dataset. a. Table showing the number of expanded (class 1) and non-expanded (class 0) STR alleles (column "Support") present in the test dataset and several metrics, including precision, recall, and F1-score in determining expanded and non-expanded alleles, accuracy, and macro-, and weighted-averages of these metrics. Macro average (Macro avg): unweighted mean of the performance metrics calculated for the Expanded and Not_Expanded class labels; and Weighted average (Weighted avg): weighted mean of the performance metrics calculated for the Expanded and Not_Expanded class labels (accounts for label imbalance). **b.** Receiver Operating Characteristics (ROC) curve with False-Positive Rate on *x*-axis and True- Positive Rate on *y*-axis showing the performance of the decision tree model on the test dataset (n=236) and Precision-recall curve showing recall on *x*-axis and precision on *y*-axis for the test dataset. **c.** Confusion matrix showing the number of expanded and non expanded alleles along the blue-shaded diagonal that were rightly-classified and three expanded alleles that were misclassified as non-expanded. **d.** Feature importances showing STR tools and their normalized (Gini) importance on *x*- and *y*-axis, respectively.

Fig S3

a

Class	Precision	Recall	F1-score	Support
1 (Expanded)	1.00	0.83	0.91	18
0 (Not_Expanded)	0.99	1.00	0.99	218
Accuracy			0.99	236
Macro avg	0.99	0.92	0.95	236
Weighted avg	0.99	0.99	0.99	236

b

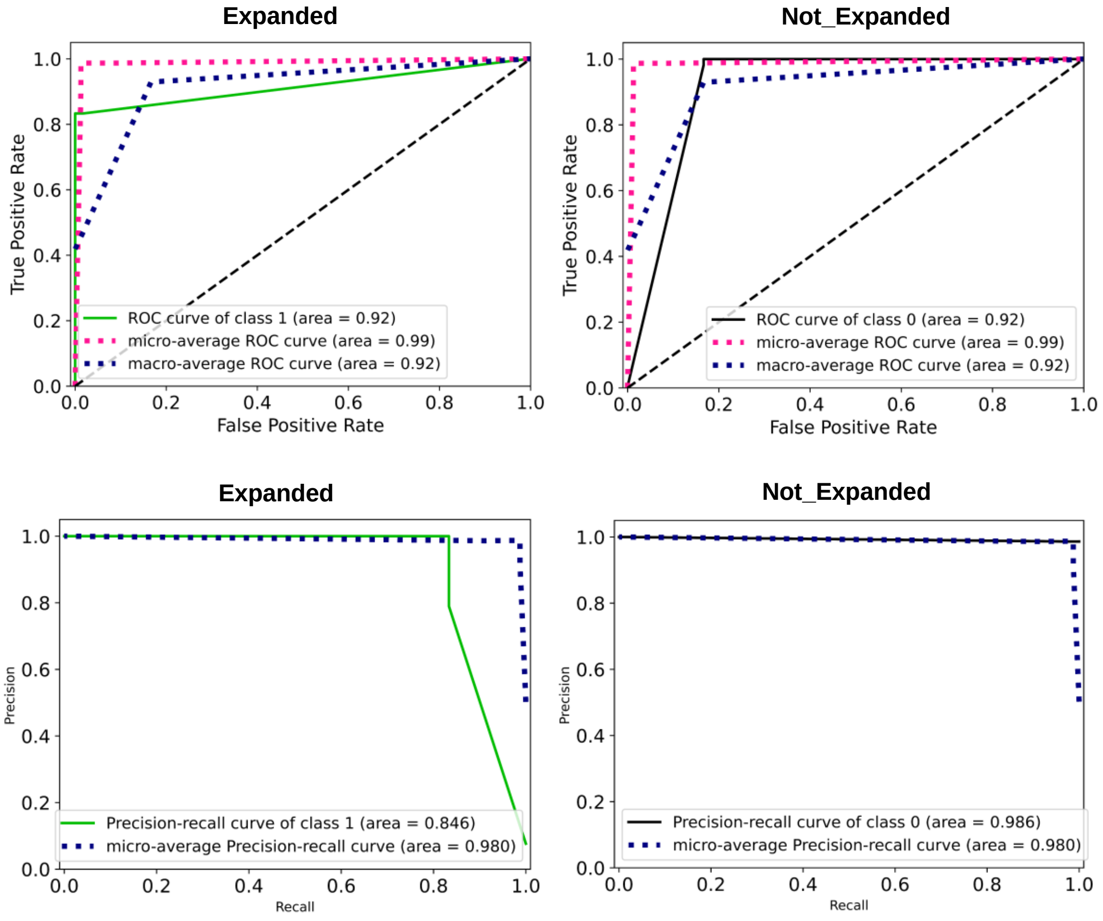

c

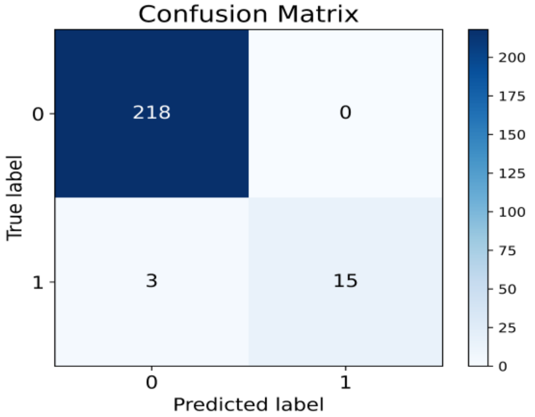

d

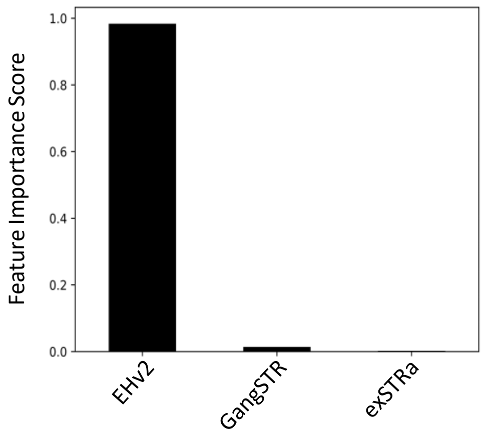

Fig S4: Decision tree model of the default analysis of BWA-aligned EGA genomes on the training dataset. Decision tree generated ~~by the classifier~~ on the training dataset (n=940). EHv3: ExpansionHunter version 3; TRED: TREDPARSE; Expanded and Not_Expanded imply the presence and absence of full-mutation, respectively.

Fig S4

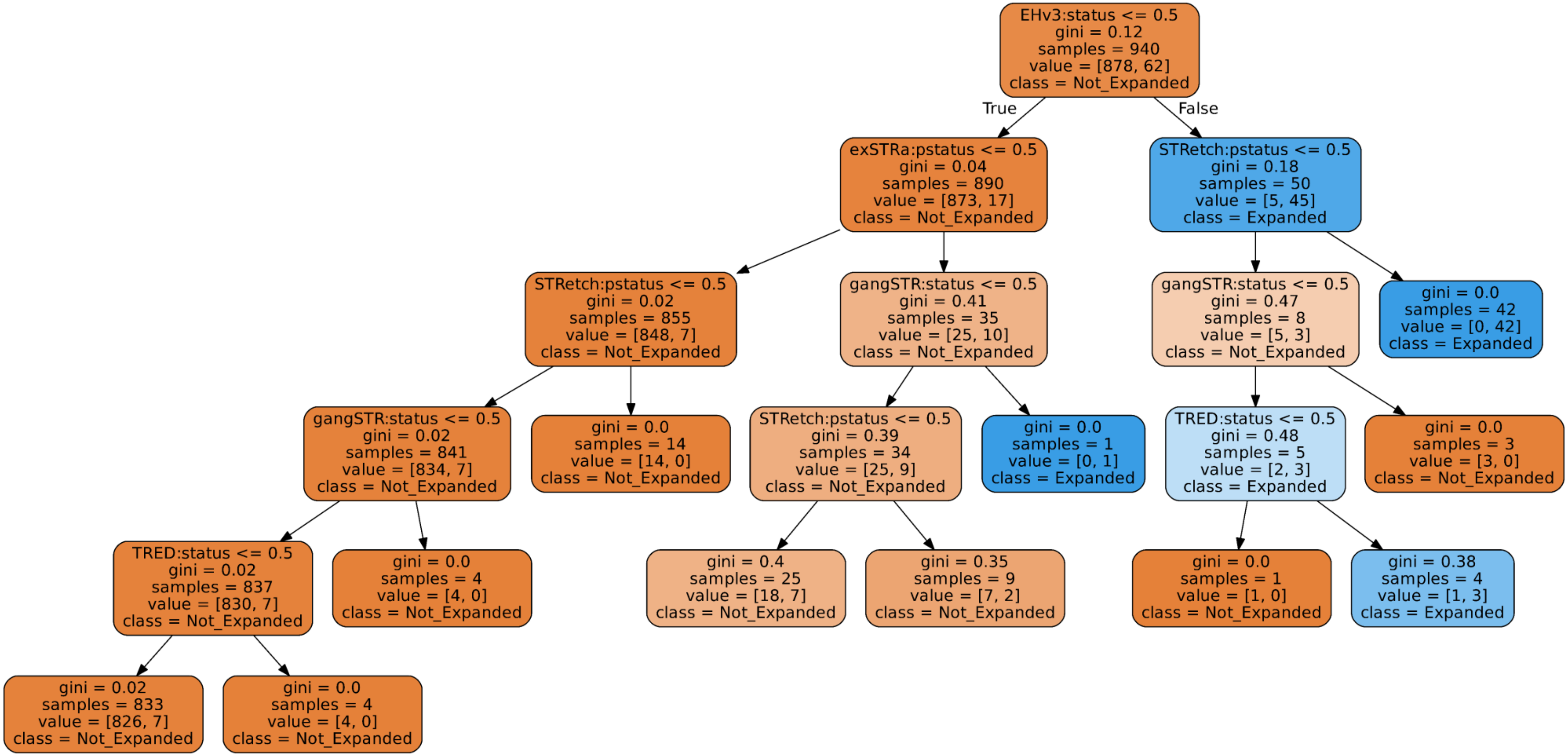

EHv3:status <= 0.5
gini = 0.12
samples = 940
value = [878, 62]
class = Not_Expanded
True
False
exSTRa:pstatus <= 0.5
gini = 0.04
samples = 890
value = [873, 17]
class = Not_Expanded
STRetch:pstatus <= 0.5
gini = 0.18
samples = 50
value = [5, 45]
class = Expanded
STRetch:pstatus <= 0.5
gini = 0.02
samples = 855
value = [848, 7]
class = Not_Expanded
gangSTR:status <= 0.5
gini = 0.41
samples = 35
value = [25, 10]
class = Not_Expanded
gangSTR:status <= 0.5
gini = 0.47
samples = 8
value = [5, 3]
class = Not_Expanded
gini = 0.0
samples = 42
value = [0, 42]
class = Expanded
gangSTR:status <= 0.5
gini = 0.02
samples = 841
value = [834, 7]
class = Not_Expanded
gini = 0.0
samples = 14
value = [14, 0]
class = Not_Expanded
STRetch:pstatus <= 0.5
gini = 0.39
samples = 34
value = [25, 9]
class = Not_Expanded
gini = 0.0
samples = 1
value = [0, 1]
class = Expanded
TRED:status <= 0.5
gini = 0.48
samples = 5
value = [2, 3]
class = Expanded
gini = 0.0
samples = 3
value = [3, 0]
class = Not_Expanded
TRED:status <= 0.5
gini = 0.02
samples = 837
value = [830, 7]
class = Not_Expanded
gini = 0.0
samples = 4
value = [4, 0]
class = Not_Expanded
gini = 0.4
samples = 25
value = [18, 7]
class = Not_Expanded
gini = 0.35
samples = 9
value = [7, 2]
class = Not_Expanded
gini = 0.0
samples = 1
value = [1, 0]
class = Not_Expanded
gini = 0.38
samples = 4
value = [1, 3]
class = Expanded
gini = 0.02
samples = 833
value = [826, 7]
class = Not_Expanded
gini = 0.0
samples = 4
value = [4, 0]
class = Not_Expanded

Fig S5: Performance metrics of the decision tree model in the default analysis of BWA-aligned EGA genomes on the test dataset. **a.** Table showing the number of expanded (class 1) and non-expanded (class 0) STR alleles (column "Support") present in the test dataset and several metrics, including precision, recall, and F1-score in determining expanded and non-expanded alleles, accuracy, and macro-, and weighted-averages of these metrics. **b.** Receiver Operating Characteristics (ROC) curve with False-Positive Rate on *x*-axis and True- Positive Rate on *y*-axis showing the performance of the decision tree model on the test dataset (n=236) and Precision-recall curve showing recall on *x*-axis and precision on *y*-axis for the test dataset. **c.** Confusion matrix showing the number of expanded and non expanded alleles along the blue-shaded diagonal that were rightly-classified and two expanded alleles that were misclassified as non-expanded. **d.** Feature importances showing STR tools and their normalized (Gini) importance on *x*- and *y*-axis, respectively.

Fig S5

a

Class	Precision	Recall	F1-score	Support
1 (Expanded)	1.00	0.89	0.94	18
0 (Not_Expanded)	0.99	1.00	1.00	218
Accuracy			0.99	236
Macro avg	1.00	0.94	0.97	236
Weighted avg	0.99	0.99	0.99	236

b

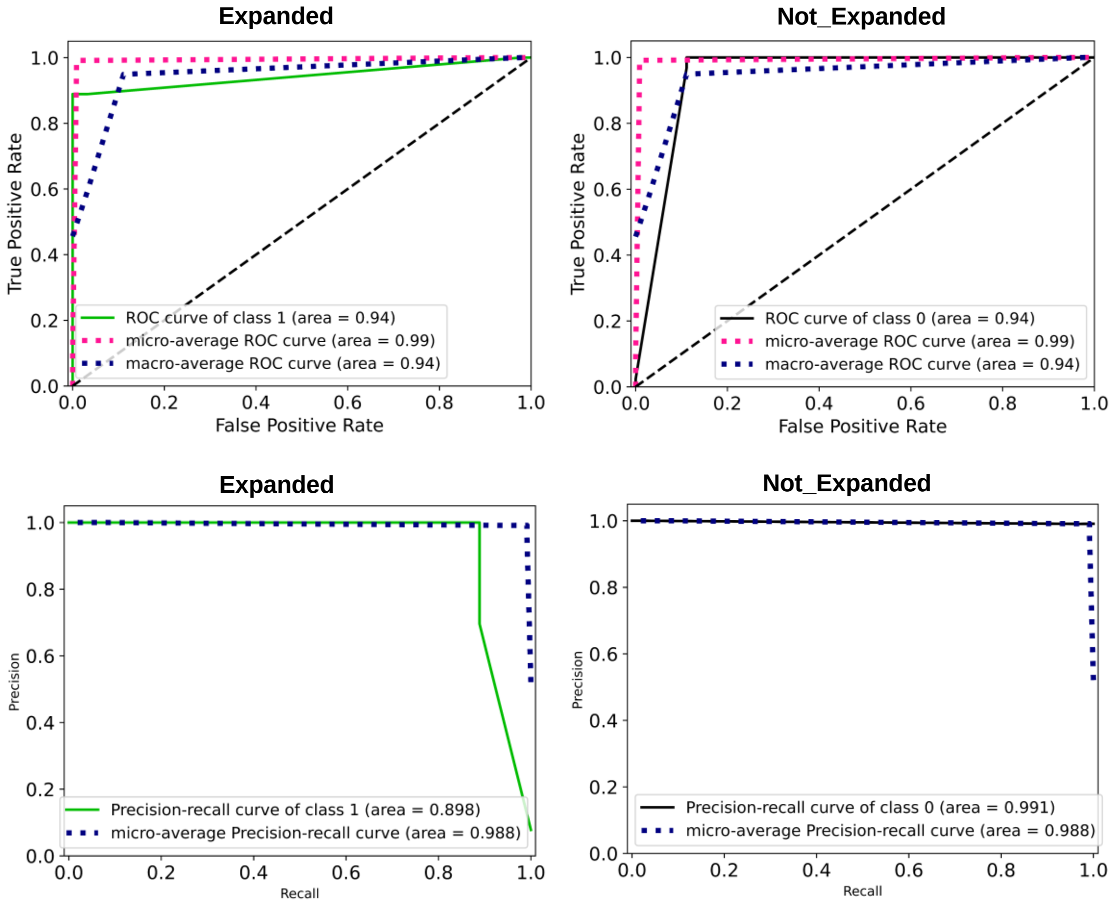

c

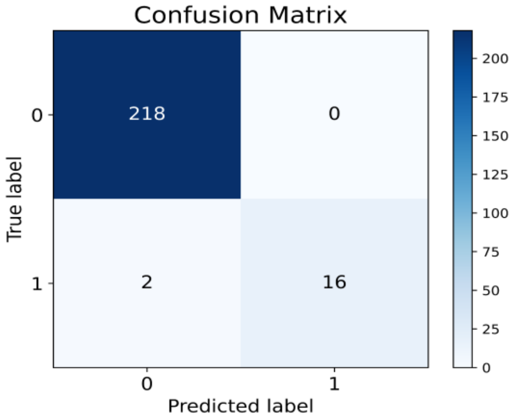

d

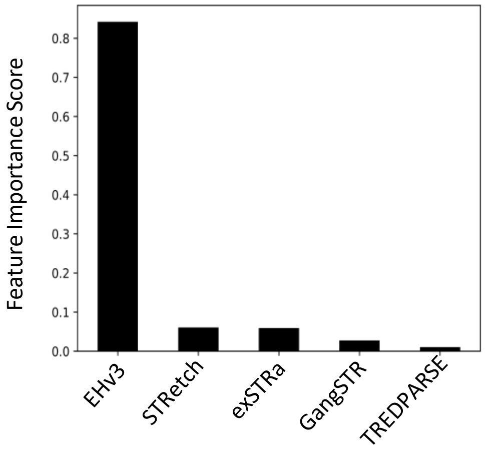

Fig S6: exSTRa plots of EGA genomes analysed with 100 controls. a. Isaac alignments. **b.** BWA alignments. ECDF (left) and t-sum (right) plots are shown.

Fig S6

a

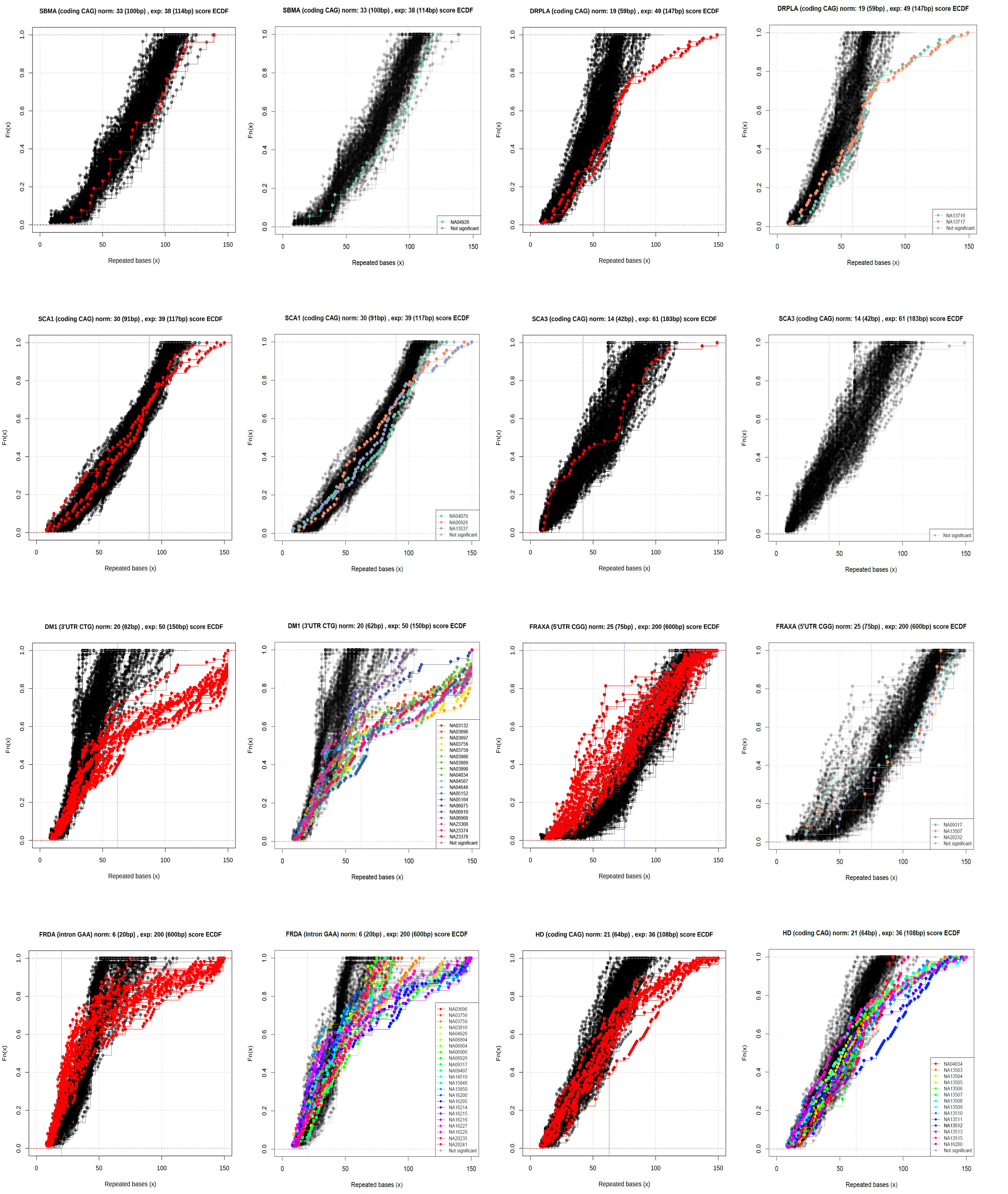
SBMA (coding CAG) norm: 33 (100bp) , exp: 38 (114bp) score ECDF
SBMA (coding CAG) norm: 33 (100bp) , exp: 38 (114bp) score ECDF
NA04926
Not significant
DRPLA (coding CAG) norm: 19 (59bp) , exp: 49 (147bp) score ECDF
DRPLA (coding CAG) norm: 19 (59bp) , exp: 49 (147bp) score ECDF
NA13716
NA13717
Not significant
SCA1 (coding CAG) norm: 30 (91bp) , exp: 39 (117bp) score ECDF
SCA1 (coding CAG) norm: 30 (91bp) , exp: 39 (117bp) score ECDF
NA04079
NA06926
NA13537
Not significant
SCA3 (coding CAG) norm: 14 (42bp) , exp: 61 (183bp) score ECDF
SCA3 (coding CAG) norm: 14 (42bp) , exp: 61 (183bp) score ECDF
Not significant
DM1 (3'UTR CTG) norm: 20 (62bp) , exp: 50 (150bp) score ECDF
DM1 (3'UTR CTG) norm: 20 (62bp) , exp: 50 (150bp) score ECDF
NA03132
NA03696
NA03697
NA03756
NA03759
NA03986
NA03989
NA03990
NA04034
NA04567
NA04648
NA05152
NA05164
NA06075
NA06910
NA06968
NA23300
NA23374
NA23378
Not significant
FRAXA (5'UTR CGG) norm: 25 (75bp) , exp: 200 (600bp) score ECDF
FRAXA (5'UTR CGG) norm: 25 (75bp) , exp: 200 (600bp) score ECDF
NA09317
NA13507
NA20232
Not significant
FRDA (intron GAA) norm: 6 (20bp) , exp: 200 (600bp) score ECDF
FRDA (intron GAA) norm: 6 (20bp) , exp: 200 (600bp) score ECDF
NA03696
NA03756
NA03759
NA03816
NA04926
NA06894
NA06904
NA06906
NA06926
NA09317
NA09497
NA14519
NA15848
NA15850
NA16200
NA16205
NA16214
NA16215
NA16216
NA16227
NA16229
NA20235
NA20241
Not significant
HD (coding CAG) norm: 21 (64bp) , exp: 36 (108bp) score ECDF
HD (coding CAG) norm: 21 (64bp) , exp: 36 (108bp) score ECDF
NA04034
NA13503
NA13504
NA13505
NA13506
NA13507
NA13508
NA13509
NA13510
NA13511
NA13512
NA13513
NA13515
NA16200
Not significant
Fn(x)
Repeated bases (x)
0.0
0.2
0.4
0.6
0.8
1.0
0
50
100
150

Fig S6

b

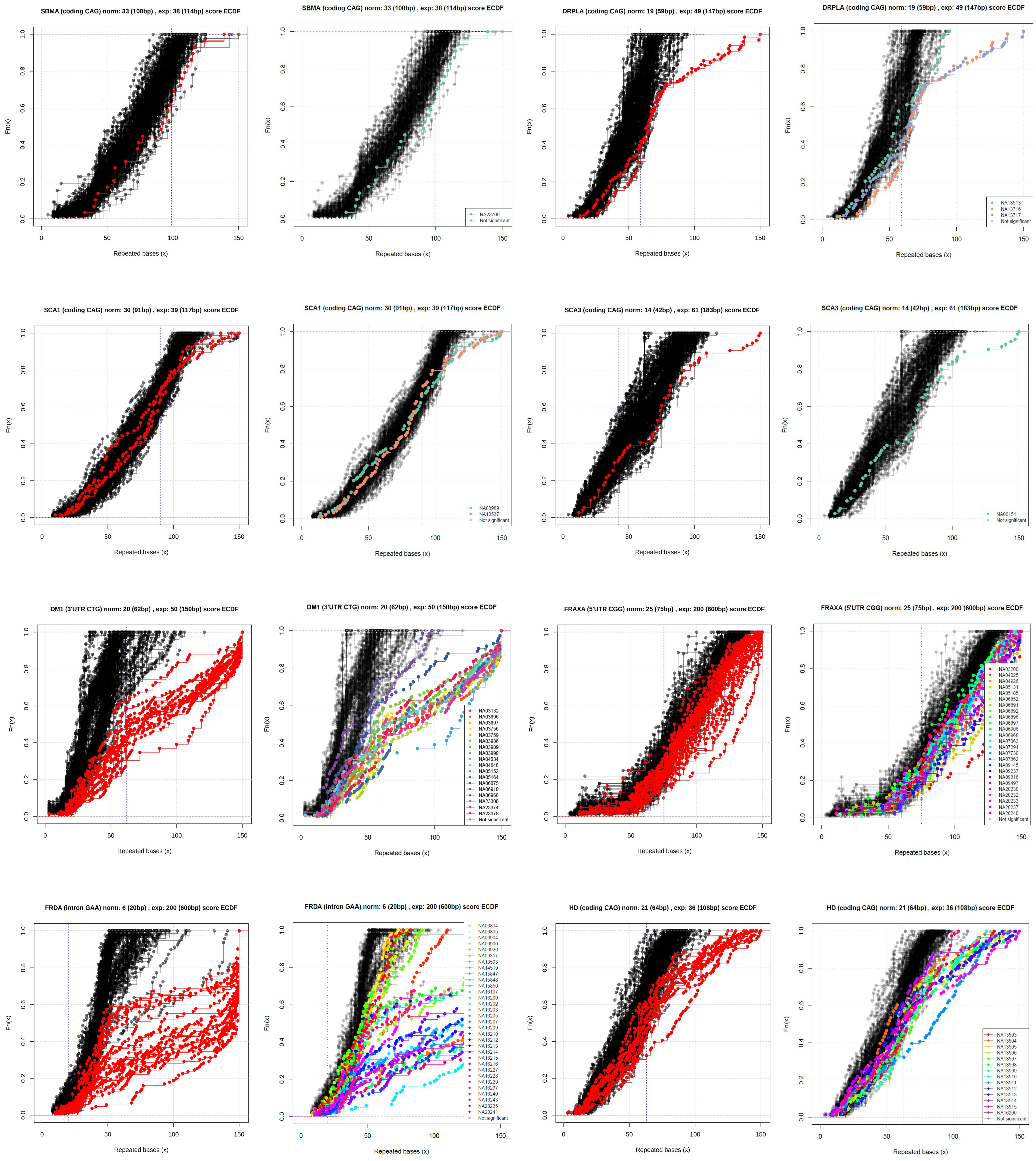
SBMA (coding CAG) norm: 33 (100bp) , exp: 38 (114bp) score ECDF
DRPLA (coding CAG) norm: 19 (59bp) , exp: 49 (147bp) score ECDF
SCA1 (coding CAG) norm: 30 (91bp) , exp: 39 (117bp) score ECDF
SCA3 (coding CAG) norm: 14 (42bp) , exp: 61 (183bp) score ECDF
DM1 (3'UTR CTG) norm: 20 (62bp) , exp: 50 (150bp) score ECDF
FRAXA (5'UTR CGG) norm: 25 (75bp) , exp: 200 (600bp) score ECDF
FRDA (intron GAA) norm: 6 (20bp) , exp: 200 (600bp) score ECDF
HD (coding CAG) norm: 21 (64bp) , exp: 36 (108bp) score ECDF
Fn(x)
Repeated bases (x)
NA23709
NA13513
NA13716
NA13717
NA03989
NA13537
NA06151
Not significant

Fig S7: Decision tree model of the modified analysis of Isaac-aligned EGA genomes on the training dataset. Decision tree generated ~~by the classifier~~ on the training dataset (n=942). EHv3: ExpansionHunter version 3; TRED: TREDPARSE; Expanded and Not_Expanded imply the presence and absence of full-mutation, respectively.

Fig S7

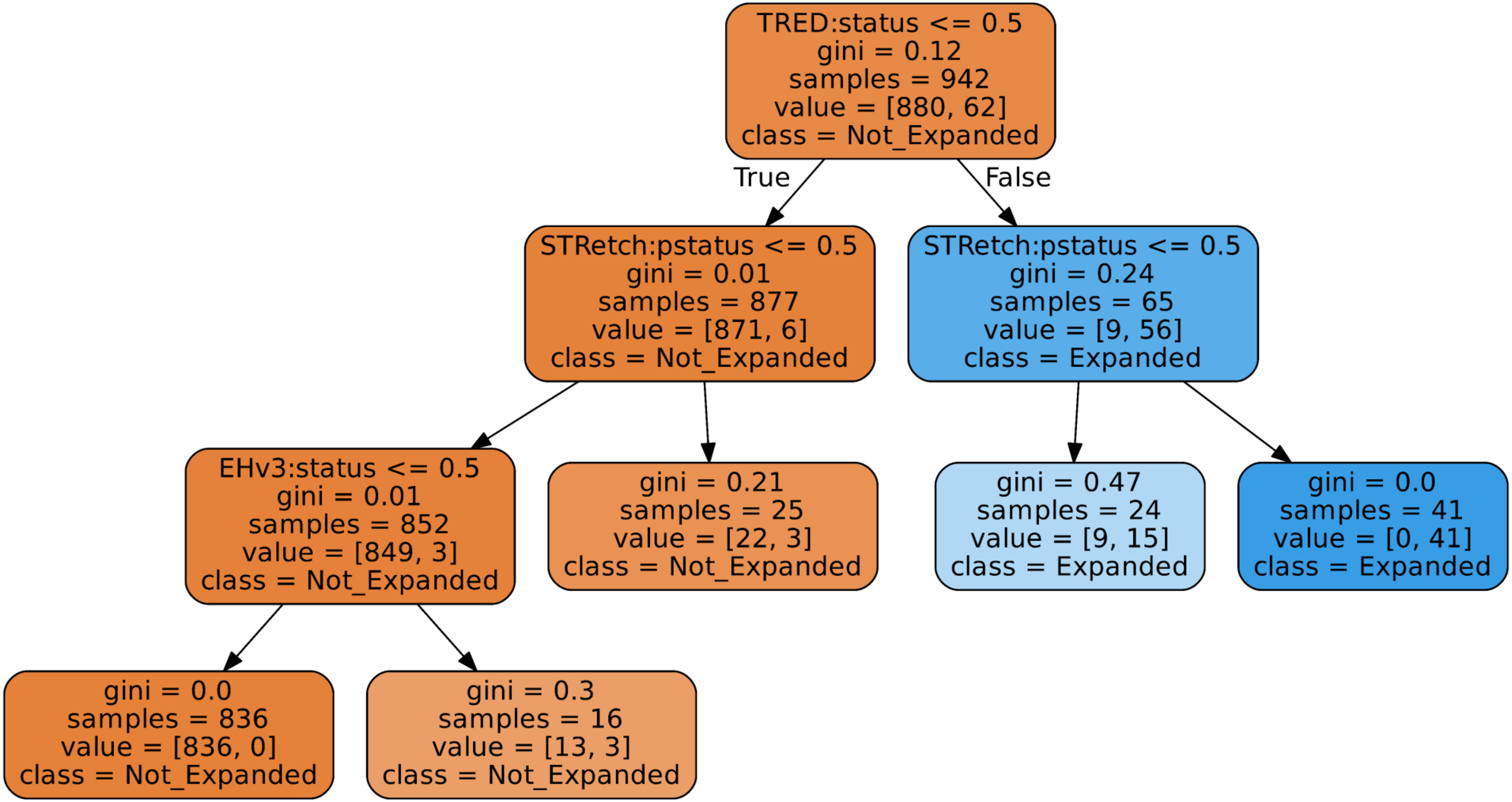
TRED:status <= 0.5
gini = 0.12
samples = 942
value = [880, 62]
class = Not_Expanded
True
False
STRetch:pstatus <= 0.5
gini = 0.01
samples = 877
value = [871, 6]
class = Not_Expanded
STRetch:pstatus <= 0.5
gini = 0.24
samples = 65
value = [9, 56]
class = Expanded
EHv3:status <= 0.5
gini = 0.01
samples = 852
value = [849, 3]
class = Not_Expanded
gini = 0.21
samples = 25
value = [22, 3]
class = Not_Expanded
gini = 0.47
samples = 24
value = [9, 15]
class = Expanded
gini = 0.0
samples = 41
value = [0, 41]
class = Expanded
gini = 0.0
samples = 836
value = [836, 0]
class = Not_Expanded
gini = 0.3
samples = 16
value = [13, 3]
class = Not_Expanded

Fig S8: Performance metrics of decision tree model in modified analysis of Isaac-aligned EGA test dataset. a. Classification report showing the number of expanded (class 1) and non-expanded (class 0) STR alleles (column "Support") present in the test dataset and several metrics, including precision, recall, and F1-score in determining expanded and non-expanded alleles, accuracy, and macro-, and weighted-averages of these metrics. **b.** Receiver Operating Characteristics (ROC) curve with False-Positive Rate on *x*-axis and True-Positive Rate on *y*-axis showing the performance of decision tree model on test dataset (n=236) and Precision-recall curve showing recall on *x*-axis and precision on *y*-axis for test dataset. **c.** Confusion matrix showing the number of expanded and non expanded alleles along the blue-shaded diagonal that were rightly-classified and two non-expanded alleles that were misclassified as expanded. **d.** Feature importances showing STR tools and their normalized (Gini) importance on *x*- and *y*-axis, respectively.

Fig S8

a

Class	Precision	Recall	F1-score	Support
1 (Expanded)	0.90	1.00	0.95	18
0 (Not_Expanded)	1.00	0.99	1.00	218
Accuracy			0.99	236
Macro avg	0.95	1.00	0.97	236
Weighted avg	0.99	0.99	0.99	236

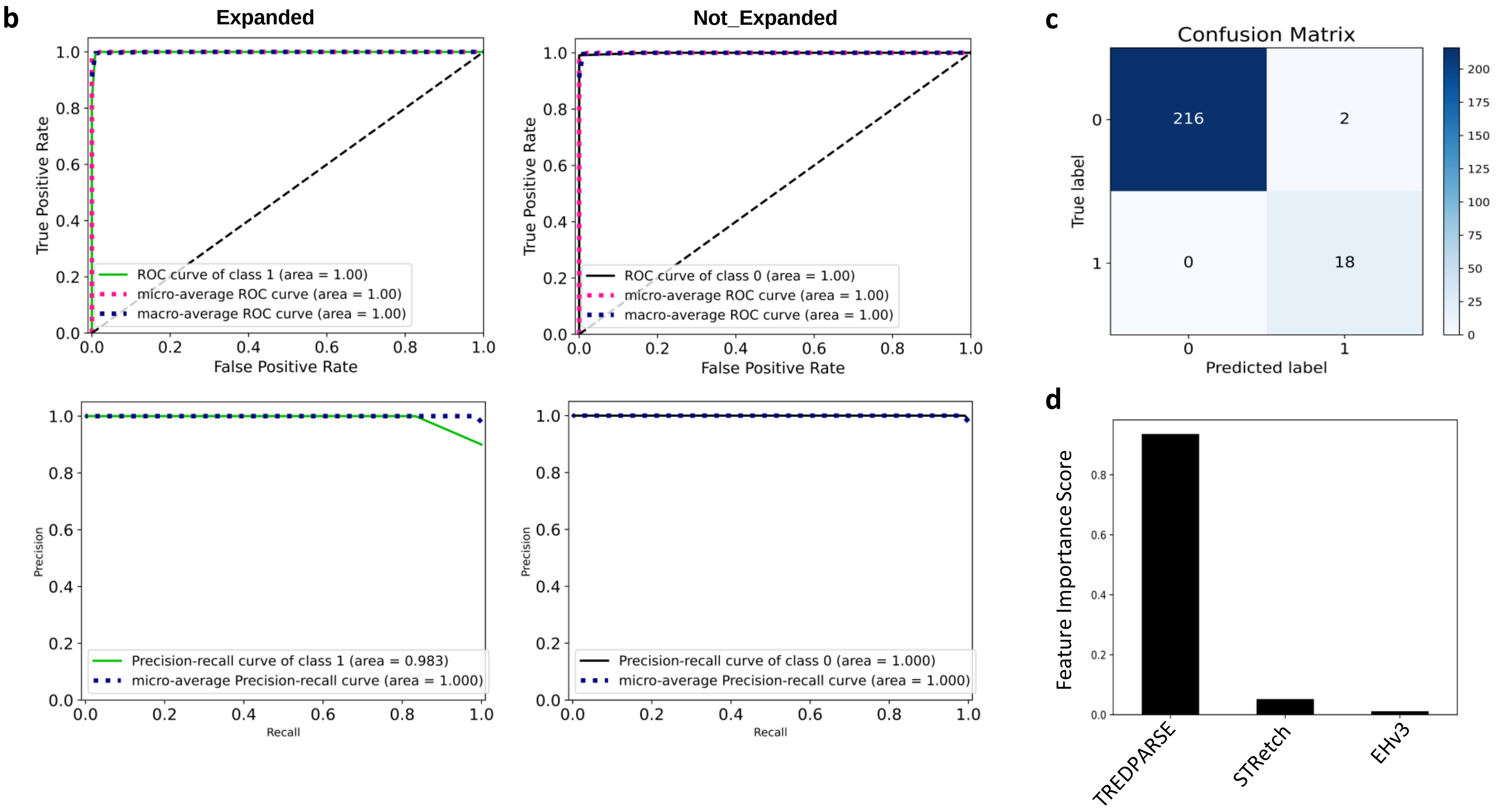

Fig S9: Allele frequency distribution of analysed disease short tandem repeat loci in the CAUSES exomes. Genotype calls of ExpansionHunter versions 2 and 3 and GangSTR were used to generate the distribution barplots. The *x*-axis shows the repeat count of the genotyped alleles and the *y*-axis shows the frequency i.e., the number of alleles with a particular repeat count. The 0 repeat alleles in the plots for X chromosome short tandem repeats (*AR*, *FMR1*, and *FMR2*) are that of hemizygous males in whom the second allele's repeat length was set to 0 and do not reflect the actual genotype calls of the tools.

Fig S9

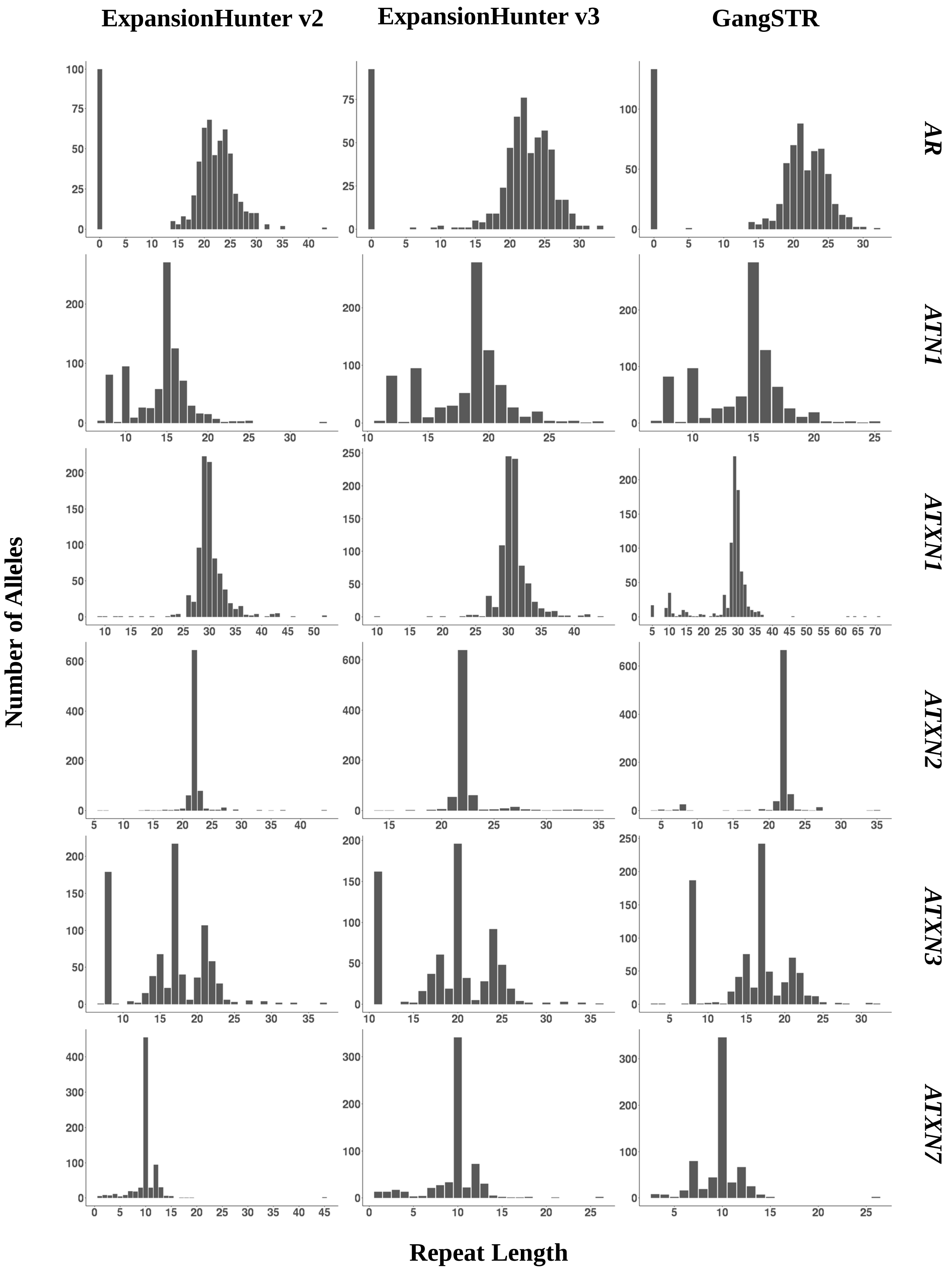
ExpansionHunter v2
ExpansionHunter v3
GangSTR
AR
ATN1
ATXN1
ATXN2
ATXN3
ATXN7
Number of Alleles
Repeat Length

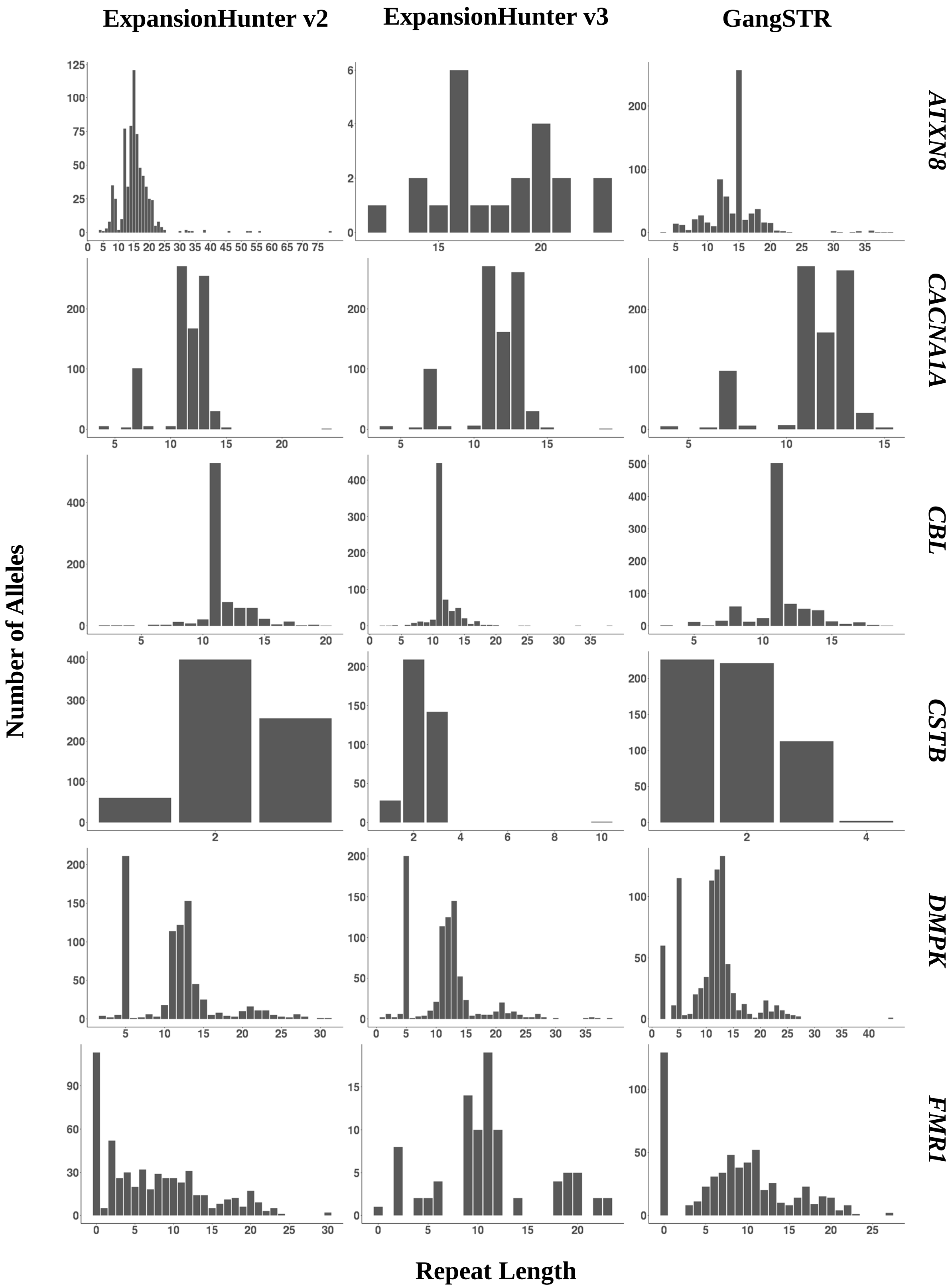
ExpansionHunter v2
ExpansionHunter v3
GangSTR
ATXN8
CACNA1A
CBL
CSTB
DMPK
FMR1
Number of Alleles
Repeat Length

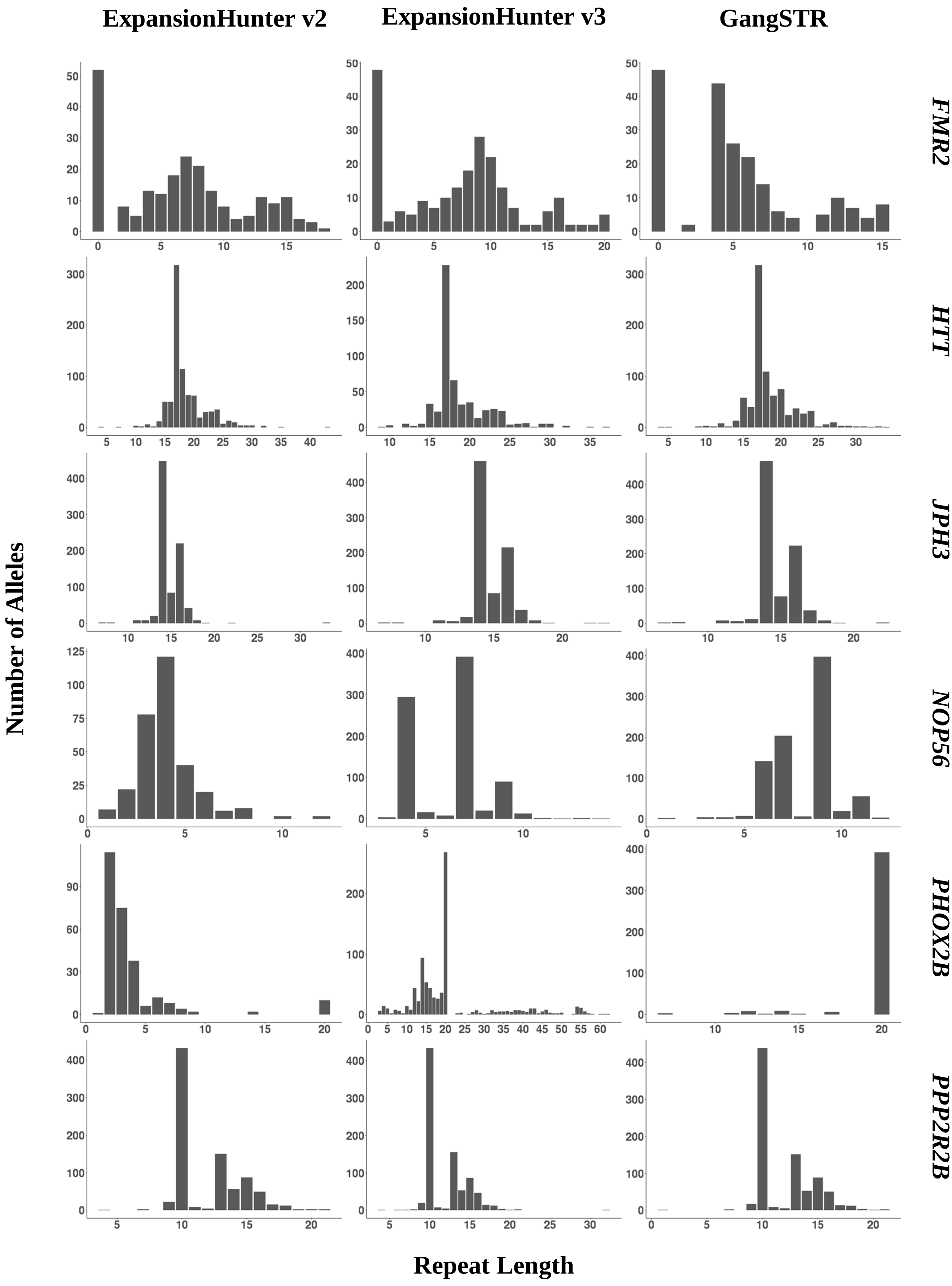
ExpansionHunter v2
ExpansionHunter v3
GangSTR
FMR2
HTT
JPH3
NOP56
PHOX2B
PPP2R2B
Number of Alleles
Repeat Length

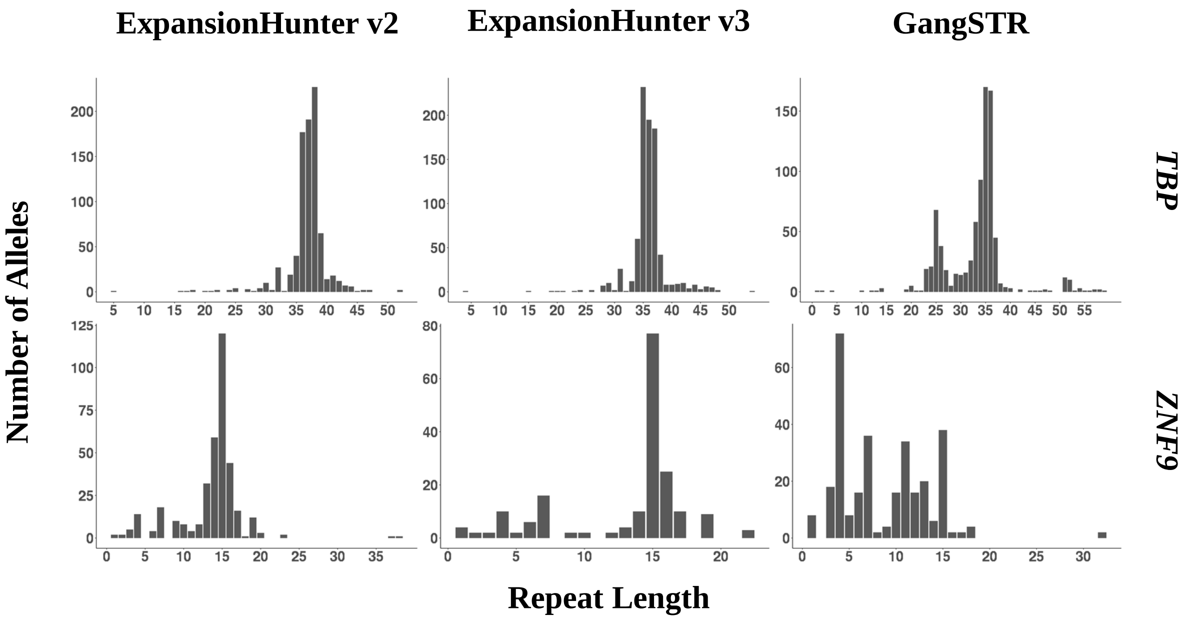

ExpansionHunter v2
ExpansionHunter v3
GangSTR
TBP
ZNF9
Number of Alleles
Repeat Length

Fig S10: Allele frequency distribution of analysed disease short tandem repeat loci in the CAUSES and IMAGINE genomes.

Fig S10

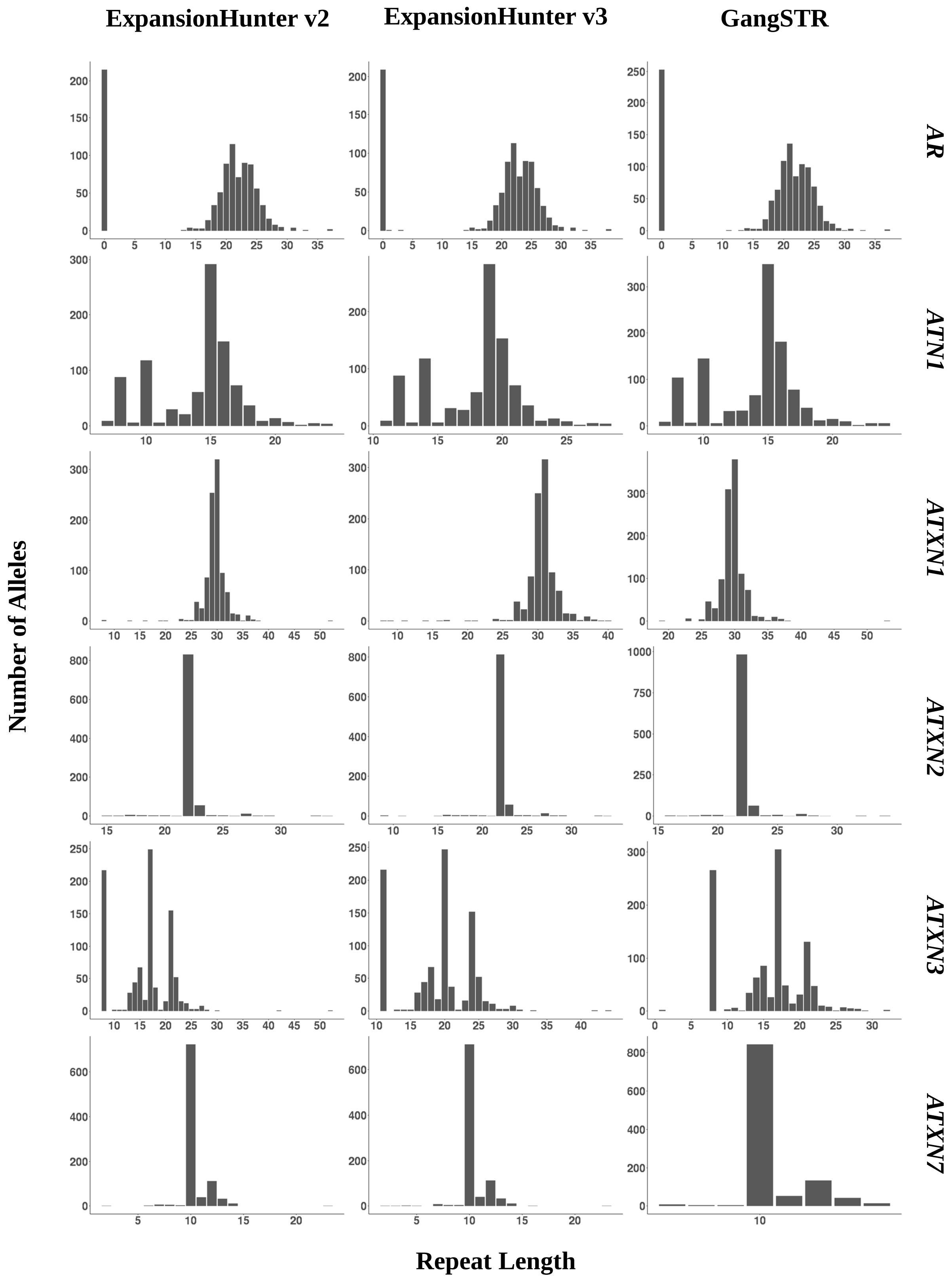
ExpansionHunter v2
ExpansionHunter v3
GangSTR
AR
ATN1
ATXN1
ATXN2
ATXN3
ATXN7
Number of Alleles
Repeat Length

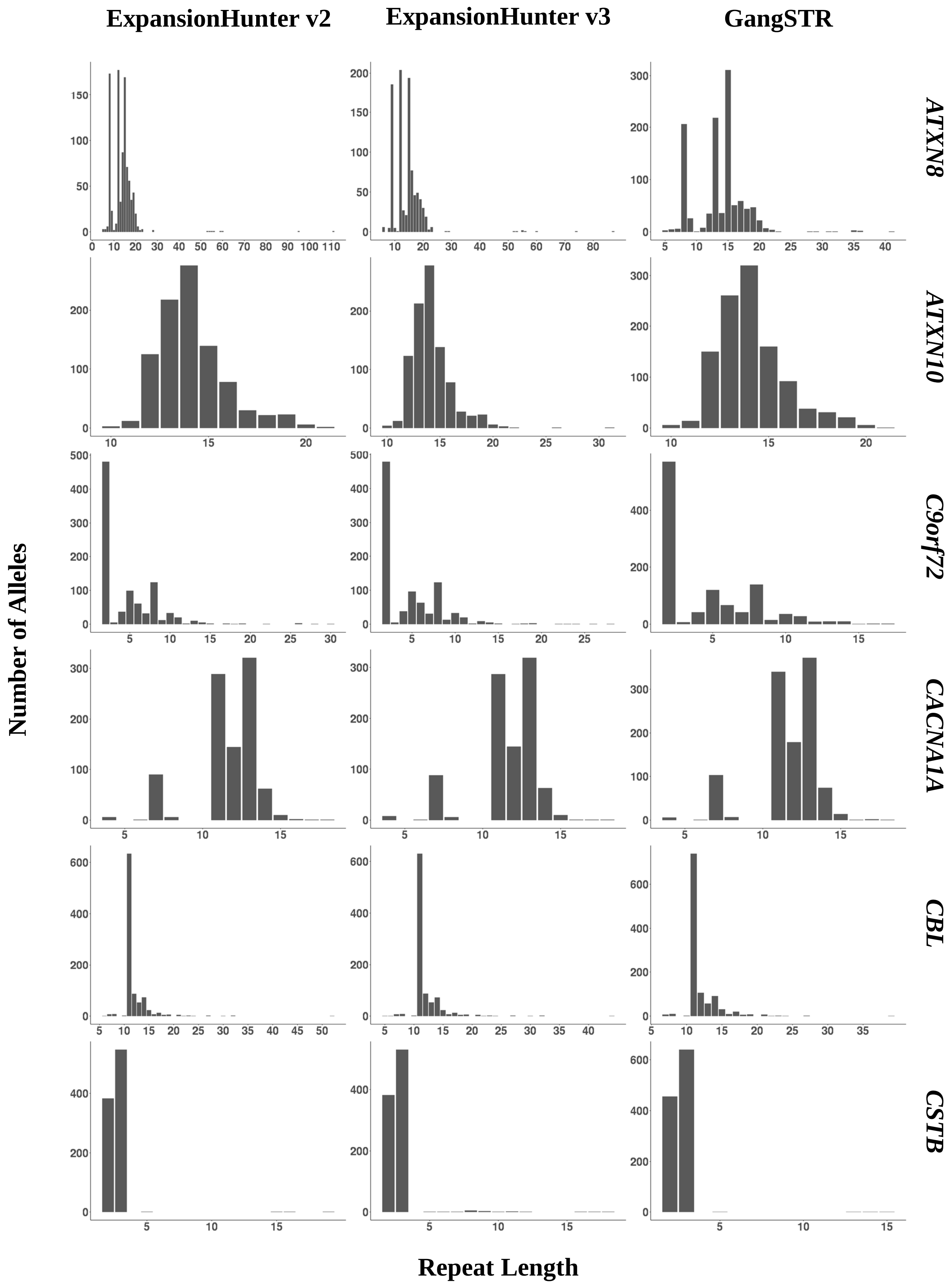
ExpansionHunter v2
ExpansionHunter v3
GangSTR
ATXN8
ATXN10
C9orf72
CACNA1A
CBL
CSTB
Number of Alleles
Repeat Length

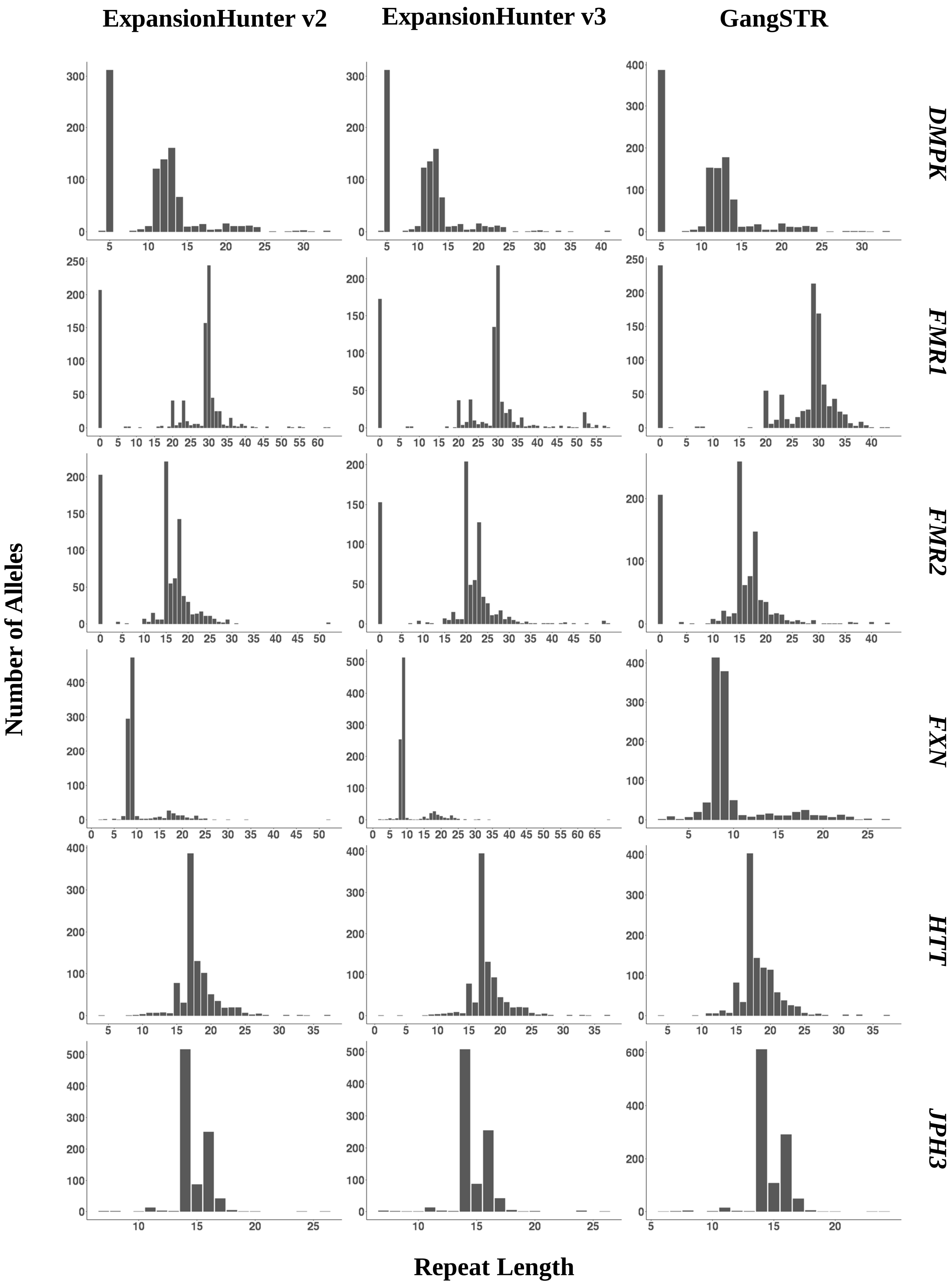
ExpansionHunter v2
ExpansionHunter v3
GangSTR
DMPK
FMR1
FMR2
FXN
HTT
JPH3
Number of Alleles
Repeat Length

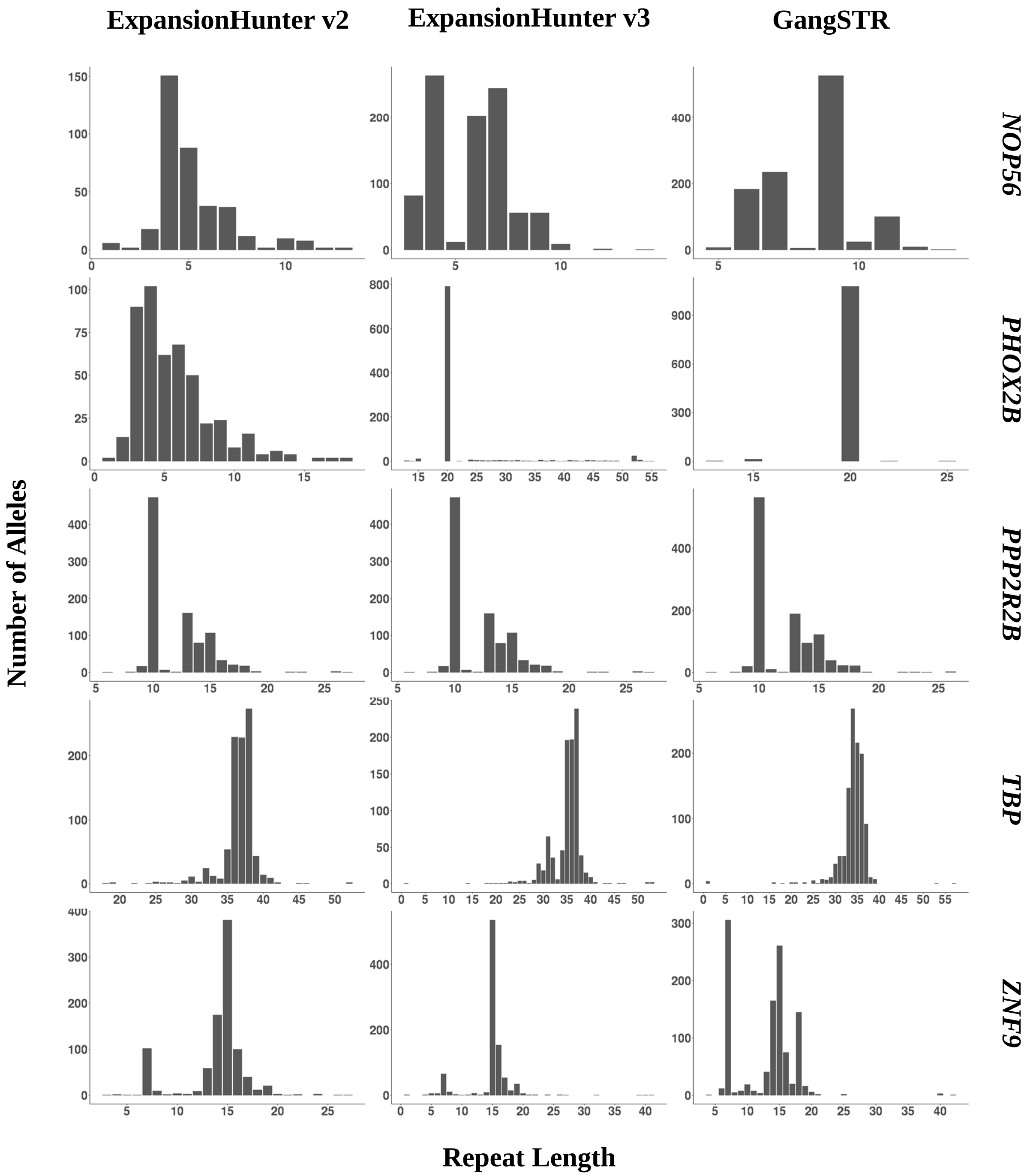
ExpansionHunter v2
ExpansionHunter v3
GangSTR
NOP56
PHOX2B
PPP2R2B
TBP
ZNF9
Number of Alleles
Repeat Length

Fig S11: Coverage and alignment statistics of Isaac- and BWA-aligned EGA genomes. samtools depth and stats were used to calculate the average coverage and alignment statistics, respectively, across the analysed ten disease short tandem repeat loci. The bar plots show the averages computed across 118 genomes (±standard error) on *y*-axis and the target short tandem repeat loci on *x*-axis.

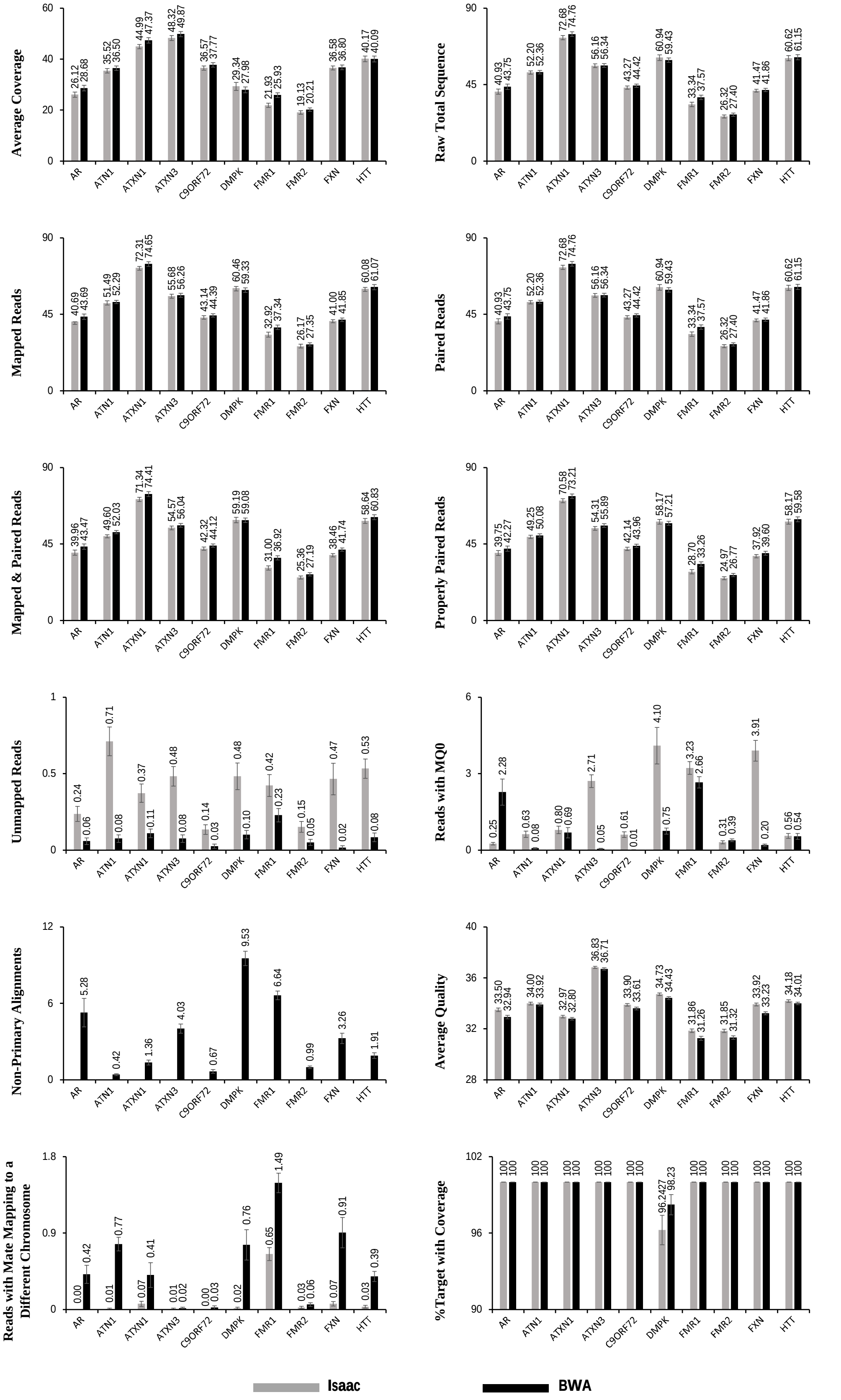

Fig S12: Analysis of the *DMPK* locus by ExpansionHunter version 2 with and without off-target sites in the EGA dataset. Results of Isaac-(top panels) and BWA-aligned (bottom panels) genomes. Scatter plots on the left show the estimated repeat lengths of the *DMPK* locus in the EGA genomes by ExpansionHunter version 2 with off-target (OT) sites (wOT) and without OT (nOT) sites alongside the consensus repeat size of known *DMPK* expansion-positive samples. The horizontal red-dotted line define the lower-bound repeat lengths of *DMPK* full-mutations (50 repeats). Plots on the right show the Pearson's correlation between estimated and consensus repeat lengths of full-mutation alleles wOT and nOT.

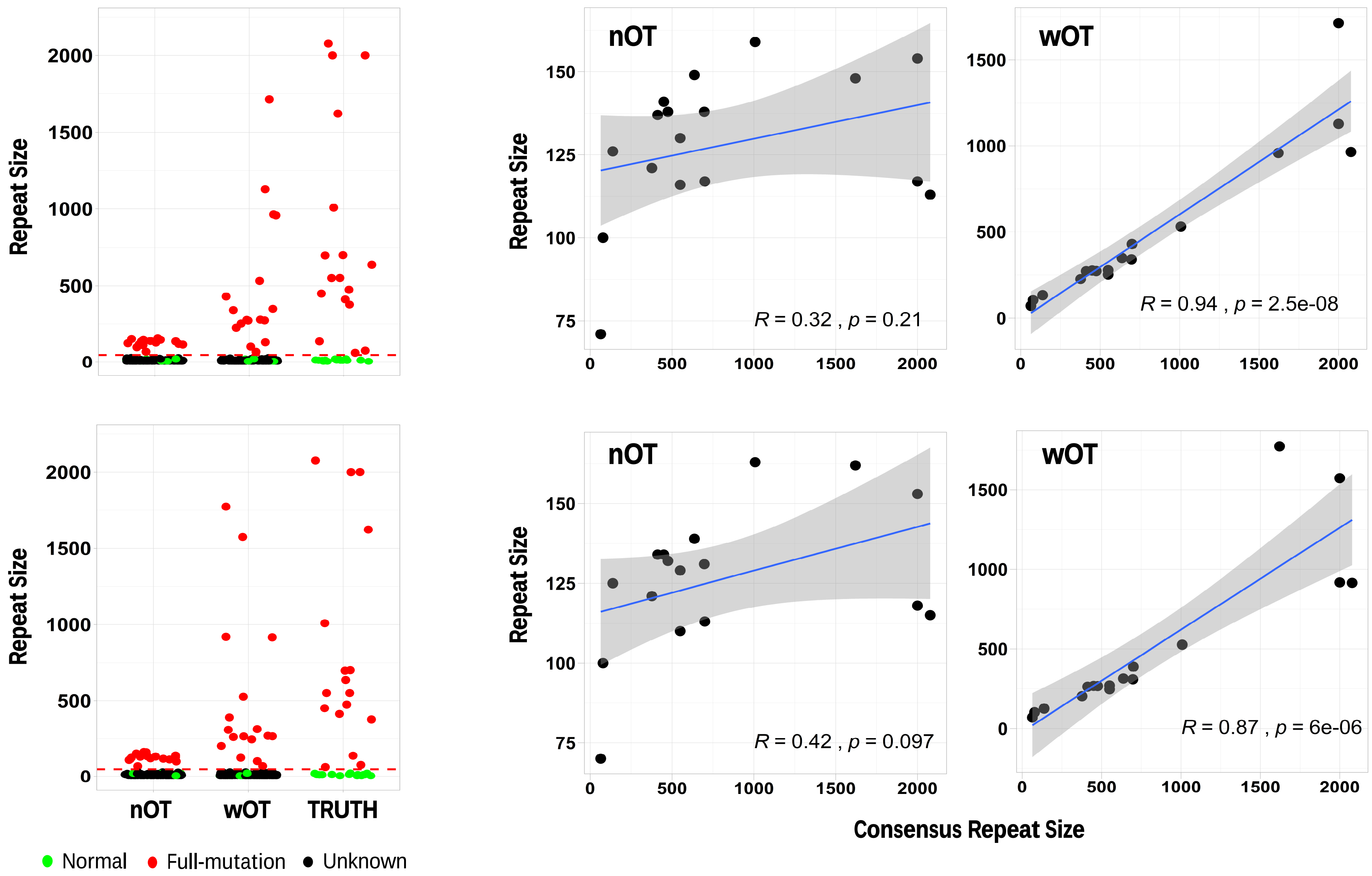

Supplement: Supplementary file 3 — Additional file 3: Fig S1: exSTRa plots of EGA and simulated genomes. Fig S2: Decision tree model of the default analysis of Isaac-aligned EGA genomes on the training dataset. Fig S3: Performance metrics of the decision tree model in the default analysis of Isaac-aligned EGA genomes on the test dataset. Fig S4: Decision tree model of the default analysis of BWA-aligned EGA genomes on the training dataset. Fig S5: Performance metrics of the decision tree model in the default analysis of BWA-aligned EGA genomes on the test dataset. Fig S6: exSTRa plots of EGA genomes analyzed with 100 controls. Fig S7: Decision tree model of the modified analysis of Isaac-aligned EGA genomes on the training dataset. Fig S8: Performance metrics of decision tree model in the modified analysis of Isaac-aligned EGA test dataset. Fig S9: Allele frequency distribution of analyzed disease short tandem repeat loci in the CAUSES exomes. Fig S10: Allele frequency distribution of analyzed disease short tandem repeat loci in the CAUSES and IMAGINE genomes. Fig S11: Coverage and alignment statistics of Isaac- and BWA-aligned EGA genomes. Fig S12: Analysis of the DMPK locus by ExpansionHunter version 2 with and without off-target sites in the EGA dataset. [file 13073_2021_932_MOESM3_ESM.pdf]
